# Remodeling of T Cell Dynamics During Long COVID Is Dependent on Severity of SARS-CoV-2 Infection

**DOI:** 10.3389/fimmu.2022.886431

**Published:** 2022-06-10

**Authors:** Milena Wiech, Piotr Chroscicki, Julian Swatler, Dawid Stepnik, Sara De Biasi, Michal Hampel, Marta Brewinska-Olchowik, Anna Maliszewska, Katarzyna Sklinda, Marek Durlik, Waldemar Wierzba, Andrea Cossarizza, Katarzyna Piwocka

**Affiliations:** ^1^Laboratory of Cytometry, Nencki Institute of Experimental Biology, Polish Academy of Sciences, Warsaw, Poland; ^2^Department of Medical and Surgical Sciences for Children and Adults, University of Modena and Reggio Emilia School of Medicine, Modena, Italy; ^3^Department of Gastroenterological Surgery and Transplantology, Central Clinical Hospital of the Ministry of Interior, Warsaw, Poland; ^4^Department of Radiology, Centre of Postgraduate Medical Education, Warsaw, Poland; ^5^Departament of Gastroenterological Surgery and Transplantology, Centre of Postgraduate Medical Education, Warsaw, Poland; ^6^Central Clinical Hospital of the Ministry of Interior, Warsaw, Poland; ^7^University of Humanities and Economics, Lodz, Poland; ^8^National Institute for Cardiovascular Research, Bologna, Italy

**Keywords:** COVID-19, long COVID, post-acute COVID-syndrome (PACS), convalescents, immune system, T cell exhaustion/senescence, inflammation resolution, full spectral cytometry

## Abstract

Several COVID-19 convalescents suffer from the post-acute COVID-syndrome (PACS)/long COVID, with symptoms that include fatigue, dyspnea, pulmonary fibrosis, cognitive dysfunctions or even stroke. Given the scale of the worldwide infections, the long-term recovery and the integrative health-care in the nearest future, it is critical to understand the cellular and molecular mechanisms as well as possible predictors of the longitudinal post-COVID-19 responses in convalescent individuals. The immune system and T cell alterations are proposed as drivers of post-acute COVID syndrome. However, despite the number of studies on COVID-19, many of them addressed only the severe convalescents or the short-term responses. Here, we performed longitudinal studies of mild, moderate and severe COVID-19-convalescent patients, at two time points (3 and 6 months from the infection), to assess the dynamics of T cells immune landscape, integrated with patients-reported symptoms. We show that alterations among T cell subsets exhibit different, severity- and time-dependent dynamics, that in severe convalescents result in a polarization towards an exhausted/senescent state of CD4+ and CD8+ T cells and perturbances in CD4+ Tregs. In particular, CD8+ T cells exhibit a high proportion of CD57+ terminal effector cells, together with significant decrease of naïve cell population, augmented granzyme B and IFN-γ production and unresolved inflammation 6 months after infection. Mild convalescents showed increased naïve, and decreased central memory and effector memory CD4+ Treg subsets. Patients from all severity groups can be predisposed to the long COVID symptoms, and fatigue and cognitive dysfunctions are not necessarily related to exhausted/senescent state and T cell dysfunctions, as well as unresolved inflammation that was found only in severe convalescents. In conclusion, the post-COVID-19 functional remodeling of T cells could be seen as a two-step process, leading to distinct convalescent immune states at 6 months after infection. Our data imply that attenuation of the functional polarization together with blocking granzyme B and IFN-γ in CD8+ cells might influence post-COVID alterations in severe convalescents. However, either the search for long COVID predictors or any treatment to prevent PACS and further complications is mandatory in all patients with SARS-CoV-2 infection, and not only in those suffering from severe COVID-19.

## Introduction

COVID-19 disease, caused by severe acute respiratory syndrome coronavirus 2 (SARS-CoV-2), has emerged at the end of 2019 and has become an ongoing pandemic, as officially declared by the WHO in March 2020 (1–3). Although SARS-Cov-2 infection and the course of COVID-19 illness is mild in a large proportion of infected individuals (4), a number of patients develop severe symptoms that can result in a fatal event (5–9). To date, there have been over 499 million globally reported cases and over 6,1 mln COVID-19–related deaths (Johns Hopkins Coronavirus Resource Center. Johns Hopkins website. https://coronavirus.jhu.edu/; updated 11^th^ April 2022).

Number of studies have already indicated that several individuals convalescent from COVID-19 suffer from the post-acute COVID-syndrome (PACS), also referred to as “long COVID”, that is characterized, among others, by long-lasting symptoms, such as fatigue, dyspnea, pulmonary fibrosis, stroke, and other cerebrovascular events, as well as cognitive and neurological dysfunctions (2, 10). This significantly affects both the quality of life and everyday activities, and is also relevant in terms of future medical treatments. Although the majority of long-term studies have concentrated on patients experiencing a severe form of COVID-19, recent data show that different types of PACS-related symptoms can be found not only in them, but also in mild and even asymptomatic cases, although in such cases, the immune landscape and possible mechanisms are far less clear (11–15). However, despite the number of studies on COVID-19, little is known about the mechanisms, nor about molecular and cellular changes in these long-term effects, as well as about the presence of possible predictors. Nevertheless, changes in the peripheral immune system, which persist long after the infection is cleared, might have potential implications for understanding symptoms and identify predictors associated with long COVID (14, 16–18). Therefore, given the scale of the worldwide infections, the long-term recovery and the integrative health-care in the future perspective, it is critical to understand molecular and cellular mechanisms of immune responses in convalescent individuals of different severity of the disease.

The immune system immediately responds to infection with SARS-CoV-2 (19), and several perturbations in individuals with severe infection have been described in detail since the earliest moments of the pandemic (20–24). Systemic inflammation and markedly high levels of proinflammatory cytokines in plasma, named “cytokine storm”, are characteristic for COVID-19 severe patients and are recognized as clinical predictors of disease outcome and mortality (25, 26). Therefore coordinated resolution of inflammation is necessary for successful recovery from acute infection, as persistent inflammatory state can lead to dysregulated immune responses (27–29).

Most severe COVID-19 convalescents have a low number of monocytes, helper T cells, memory B cells and proliferating lymphocytes over time (30, 31). Several reports showed that COVID-19 convalescence may be long and characterized by dysregulation of adaptive immunity, regarding specific CD4+ and CD8+ T cells, that express exhaustion markers for months after symptom onset (14, 32). Those alterations are associated with diminished immune activation and proinflammatory signaling (including IFN-γ, TNF-α, IL-2, IL-6) (26, 27). Finally, plasma level of inflammatory proteins, including IFN-γ, IL-6, and TNF-α, which is elevated during acute infection (26, 33), resolves over time in patients who fully recover after severe COVID-19, but remains elevated in patients with ineffective resolution of inflammation, leading to progression into persistent chronic inflammation (31). However, long-lasting studies of the immune landscape upon COVID-19 recovery are still not complete and crucial questions remain about the long-term immune responses due to COVID-19 of different severity. One of the open questions concerns the role of the severity of the infection in causing alterations in functionality and activity of immune cells in convalescent patients, and in other post-COVID-19 long-lasting symptoms.

Here, we describe our longitudinal studies of convalescent patients who had experienced a mild, moderate or severe COVID-19, who were interrogated at two time-points (up to 3 and 6 months from the diagnosis), to assess the dynamics of immune changes of post-COVID-19. Using classical and spectral cytometry, followed by unsupervised analysis, we report an in-depth characterization of phenotypic and functional remodeling of different T cell subsets during recovery from COVID-19. Long-lasting immune changes are then interpreted taking into account T cell polyfunctionality and the levels of plasmatic inflammatory markers.

Finally, we discuss whether the observed changes are related to the development of PACS symptoms, depending on the severity of the disease. The immune spectrum and T cell polyfunctionality undergo significant dynamics during COVID-19 recovery, which result in the very different landscapes and distinct convalescent immune states at 3 or 6 months after the infection. We also describe how several immune changes and PACS symptoms are severity-dependent, being more frequent in severe convalescents, but also appearing in individuals who recovered from mild and moderate COVID-19.

## Methods

### Ethics Statement

The study protocol was approved by the ethics committee of the Central Clinical Hospital of the Internal Affairs and Administration Ministry in Warsaw (Decision No 151/2020) with informed consent of enrolled individuals. The study was performed in accordance with the latest version of the Declaration of Helsinki and the guidelines for good clinical practice.

### Stratification of COVID-19 Patients

A total of 59 male COVID-19 recovered patients between 27-64 years of age were included in the study and divided into three age-matched groups according to the severity of disease measured by the size of lung lesions confirmed by computed tomography scan and the type of applied oxygen therapy. All patients were hospitalized over the period of 25th June - 3rd November 2020 or 13th March – 23rd April 2021 at the Central Clinical Hospital of the Ministry of Interior and Administration in Warsaw. In all cases, the SARS-CoV-2 infection was confirmed by positive RT-PCR test and indicated a day of diagnosis (genotyping of viral variant was not performed; however at that time the alpha variant was dominant in Poland while the first cases of delta variant have been identified in June 2021). Patients with severe COVID-19 symptoms were selected based on the coverage of lung parenchyma with ground-glass opacities (GGO) at the range of 20 – 83% and treatment by active or passive high-flow oxygen supply (>15 l/min). Patients with moderate COVID-19 symptoms had GGO occupying 5 – 19% of lung parenchyma and were treated with low-flow oxygen supply (≤15 l/min). Patients with mild COVID-19 symptoms had GGO below 5% of lung parenchyma and received low-flow or no oxygen supply during hospitalization. Dexamethasone was applied during hospitalization to 19 of severe (out of 22), 13 of moderate (out of 21) and 6 of mild (out of 16) patients, following the clinical recommendations, and any immunomodulatory drug has not been applied. The age-matched group of 13 men without medical history of any coronavirus infections and no IgG antibodies against SARS-CoV-2 S1/S2 in the peripheral blood served as a reference group (healthy). Patients were interrogated at two time-points: time-point I up to 3 months and time-point II up to 6 months from the diagnosis. None of the analyzed convalescents or healthy controls underwent anti-COVID-19 vaccine before blood donation, none of the convalescents included in the studies was re-infected with SARS-CoV-2 till the follow-up interview. The detailed information is present in **Supplementary Table 1**.

### Blood Collection and Isolation of Mononuclear Cells

30 mL of peripheral blood was collected from each patient in vacuettes containing ethylenediamine-tetraacetic acid. Blood was immediately processed. Plasma was collected after centrifugation of whole blood at 150 rcf for 15 minutes at RT. Collected supernatant (*i.e.* plasma) has been transferred to a clean 15 mL conical tube and centrifuged at 2120 rcf for 15 minutes at + 4°C to deplete platelets and residual cells. Following centrifugation, plasma was aliquoted and stored at - 80°C until use. Isolation of peripheral blood mononuclear cells (PBMC) was performed using ficoll-hypaque (Lymphoprep, Stem Cell) according to standard procedures (34). PBMC were then stored in cryovials at the concentration of 4–10x10^6^/mL in liquid nitrogen in fetal bovine serum supplemented with 10% dimethyl sulfoxide. To avoid and minimize the so-called “batch effect” and variability due to processing across long timespan and to increase data quality and reproducibility, 12 (for cytokine staining) or 24 (for phenotyping) samples were thawed simultaneously, stained using one master antibodies cocktail and acquired with the same instrument settings. Measurements were taken from individual patients; in the case of plasma, each measurement was performed in duplicate and only the mean was considered and shown.

### Quantification of Cytokine and Antibody Levels in Blood Plasma

The plasma levels of 13 molecular species was quantified using a BioLegend platform (LEGENDplex™ HU Essential Immune Response Panel (13-plex), BioLegend) for the simultaneous detection of the following molecules: IL-4, IL-2, CXCL10 (IP-10), IL-1β, TNF-α, CCL2 (MCP-1), IL-17A, IL-6, IL-10, IFN-γ, IL-12p70, CXCL8 (IL-8), TGF-β1, according to the manufacturer’s instruction. The level of IgG and IgM antibodies against SARS-CoV-2 was measured using quantitative chemiluminescence immunoassay according to the manufacturer’s instruction (DiaSorin).

### T Cell Immunophenotype by Polychromatic Full Spectrum Flow Cytometry

Thawed PBMCs were washed with RPMI 1640 supplemented with 10% fetal bovine serum and 1% each of l-glutamine, sodium pyruvate, nonessential amino acids, antibiotics, 0.1 M HEPES, 55 μM β-mercaptoethanol and 0.02 mg/ml DNAse. After the second washing step using PBS, PBMCs were counted and stained with viability dye Live/Dead Blue (Thermo Fisher Scientific) and a panel of 24 fluorescent mAbs for surface staining: CD45-Krome Orange, CD3-APC-AF750, CD4-cFluor 584, CD8-BV510, CD127-APC-R700, CD25-BV421, CD45RA-BUV395, CCR7-BV785, CD27-PECy7, CD28-BUV737, CD38-APCFire810, CD57-Pacific Blue, HLA-DR-BUV805, CD95-PECy5, PD1-BV650, CCR6-BV711, CCR4-BB700, CD161-PerCP, CD73-BUV496, ICOS-BUV563, BTLA-BUV661, CCR8-PE-Dazzle 594, CD39-PECy5.5, TIGIT-BV605. Cells were washed twice with PBS, and fixed/permeabilized with eBioscience Foxp3 Transcription Factor Staining Buffer Set (Thermo Fisher Scientific). Then cells were stained with additional mAbs for intracellular staining: Foxp3-APC, Helios-FITC, RORγt-PE. Along with side and forward scatter signals, signals were obtained from all the fluorochrome-labeled mAbs. A minimum of 1,000,000 cells per sample were acquired on an Cytek Aurora flow cytometer (Cytek Biosciences). For optimal unmixing of results, for less abundant and dim markers reference controls have been prepared with the use of UltraComp eBeads Compensation Beads (Thermo Fisher Scientific) - CD25-BV421, PD1-BV650, CCR6-BV711, CCR4-BB700, CD161-PerCP, CD73-BUV496, ICOS-BUV563, BTLA-BUV661, CCR8-PE-Dazzle 594, CD39-PECy5.5, TIGIT-BV605, Foxp3-APC, Helios-FITC, RORgt-PE. In case of abundant surface markers, the single staining has been done on cells (PBMCs) - CD45-Krome Orange, CD3-APC-AF750, CD4-cFluor584, CD8-BV510, CD127-APC-R700, CD45RA-BUV395, CD27-PECy7, CD28-BUV737, CD38-APCFire810, CD57-Pacific Blue, HLA-DR-BUV805, CD95-PECy5. All antibodies used are presented in **Supplementary Table 3**. Analysis of cellular phenotypes was performed using FlowJo software (Becton Dickinson).

### *In Vitro* Stimulation and Intracellular Staining of Cytokines and Foxp3 Using Full Spectrum Cytometry

For functional assays on cytokine production by T cells, thawed isolated PBMCs were cultured overnight in RPMI 1640 supplemented with 10% fetal bovine serum and 1% each of L-glutamine, sodium pyruvate, nonessential amino acids, antibiotics, 0,1 M HEPES, 55 μM β-mercaptoethanol. After the resting step, cells were counted and stimulated for 16 h at 37°C in a 5% CO2 atmosphere with anti-CD3/CD28 (1 μg/mL) in the U-bottom 96-well plate containing complete culture medium. For each sample, at least 2x10^6^ (2 - 4 x10^6^) cells were left unstimulated as negative control, and at least 2 million (2 - 4 mln) cells were stimulated. All samples were incubated with a protein transport inhibitor containing brefeldin A (GolgiPlug, BD), monensin (GolgiStop, BD) and previously titrated concentration of CD107a-AF488. After stimulation, cells were stained with eFluor 455UV fixable viability dye (Thermo Fisher Scientific) and surface mAbs recognizing CD4-AF700, and CD8-APC-Cy7 (BioLegend). Cells were washed with PBS, and fixed and permeabilized with the eBioscience Foxp3/Transcription Factor Staining Buffer Set (Thermo Fisher Scientific) for detection of intracellular/nuclear antigens. Cells were next stained with previously titrated mAbs recognizing CD3-BUV496 (Becton Dickinson), Foxp3 PE (Thermo Fisher Scientific), TGF-β BV421 (Becton Dickinson), IL-17-PE-Cy7, TNF-α-BV605, IFN-γ-BV510, IL-2-APC, or granzymeB-PerCP-Cy5.5 (Biolegend). All antibodies used are presented in **Supplementary Table 3**. Then, a minimum of 700,000 cells per sample were acquired on the Cytek Aurora cytometer (Cytek Bioscience). Spectral unmixing has been done using UltraComp eBeads Compensation Beads (Thermo Fisher Scientific) stained separately with each individual antibody. Samples were analyzed using SpectroFlo (Cytek Bioscience) software by standard gating to eliminate aggregates and dead cells, and to identify CD3+ T cells divided into CD4+ T cells and CD8+ T cells. Additionally among CD4+ cells, regulatory T cells (Tregs) have been distinguished on the basis of Foxp3 transcription factor expression. In each group of cells the levels of above mentioned cytokines have been measured and analyzed. Manual analysis of populations producing individual cytokines was performed using SpectroFlo software and GraphPad Prism for statistics and visualization. Statistics has been done using one-way ANOVA test (* p ≤ 0,05; ** p ≤ 0,01; *** p ≤ 0,001), data are shown as Mean ± SD.

### Representation of High-Parameter Flow Cytometry (Phenotypes)

Compensated and cleaned by FlowAI Flow Cytometry Standard (FCS) 3.0 files were imported into FlowJo software version 10 (Becton Dickinson, San Josè, CA) and preprocessed by removing damaged cells and doublets. Then were selected live, undamaged CD45+CD3+CD4+ subpopulation (CD4+ T cell subset), CD45+CD3+CD8+ (CD8+ T cell subset) and CD45+CD3+CD4+CD25+CD127- (Treg cell subset). Collected subsets were normalized using cytoNorm and gaussNorm methods. All subsets were downsampled (35 000 cells per sample for CD4+ and CD8+ T cell subset and 3 000 cells for Treg subset). All downsampled cells were exported for further analysis in R using Bioconductor libraries CATALYST (version 1.16.2) and diffcyt (version 1.12.0). The data were transformed using arcsine cofactor to make distributions more symmetric and to map them to the comparable range of expression. Outliers samples were filtered out. The cell population identification was performed through unsupervised clustering using the FlowSOM (version 2.0.0) algorithm (K= 20 for CD4+ T and CD8+ T and 6 for Treg cells subset). 2D visual representation was performed applying Uniform Manifold Approximation and Projection (UMAP) on 1000 cells per sample. Then, clusters with similar distribution were merged. Statistical analysis was performed using generalized linear mixed models (GLMM) applying as FDR cutoff = 0.05.

### Representation of High-Parameter Flow Cytometry (Cytokines)

Compensated and cleaned by FlowAI Flow Cytometry Standard (FCS) 3.0 files were imported into FlowJo software version 10 (Becton Dickinson, San Josè, CA) and preprocessed by removing damaged cells and doublets. Then were selected live, undamaged CD3+CD4+ subpopulation (CD4+ T cell subset), CD3+CD8+ (CD8+ T cell subset) and CD3+CD4+FoxP3+ (Treg cell subset). All subsets were downsampled (40 000 cells per sample for CD4+ and CD8+ T cell subset and 3 400 cells for Treg subset). All downsampled cells were exported for further analysis in R using Bioconductor libraries CATALYST (version 1.16.2) and diffcyt (version 1.12.0). The data were transformed using arcsine cofactor to make distributions more symmetric and to map them to the comparable range of expression. Outliers samples were filtered out. The cell population identification was performed through unsupervised clustering using the FlowSOM (version 2.0.0) algorithm (K= 30). 2D visual representation was performed applying Uniform Manifold Approximation and Projection (UMAP) on 1000 cells per sample. Then, clusters with similar distribution were merged. Statistical analysis was performed using generalized linear mixed models (GLMM) applying as FDR cutoff = 0.05.

### Statistical Analysis

Statistical analyses were performed using Prism 6.0 (GraphPad Software Inc, la Jolla, USA). Quantitative variabilities were compared using a two-sided Mann-Whitney test, non-parametric T-test, or one-way ANOVA test, as indicated. For unsupervised analysis, the statistical analysis of grouped data (with non-normal distribution) was performed using generalized linear mixed models (GLMM) applying FDR cutoff = 0.05. Data are presented as individual values, means and standard errors of the mean. * p<0,05; ** p<0,01; *** p<0,001; **** p<0,0001.

## Results

### Characteristics of the Study Participants

**Figure 1:**
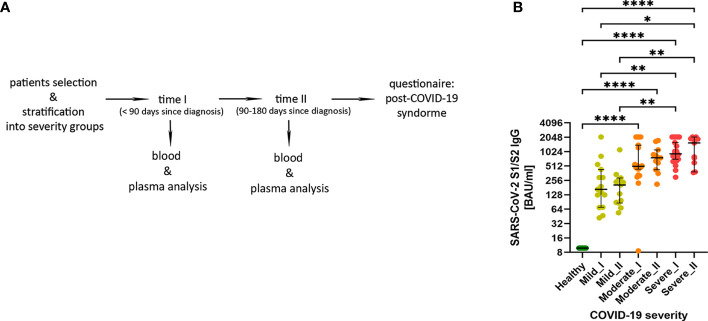
**(A)** The experimental pipeline. **(B)** IgG antibodies against SARS-Cov-2 S1/S2 antigens in COVID-19 convalescent individuals. Type of severity (mild, moderate and severe) as well as time-points (I or II) are indicated. Data shown in the log2 scale represent individual values, median +/- 95% CI (confidence interval). Statistics analysis by non-parametric T-test, *p ≤0,05; **p ≤ 0,01; ***p≤ 0,001; ****p≤ 0,0001.

We have enrolled a total of 59 patients (all male) at the age of 27-64, who have recovered from COVID-19 disease. All patients were diagnosed with SARS-CoV-2 infection based on a positive RT-PCR test from a nasopharyngeal swab specimen. If necessary, lung pneumonia was evaluated by a CT scan. Depending on the disease course of COVID-19, no oxygen supply or the therapy including low-flow oxygen supply (≤15 l/min; 51 patients) or high-flow oxygen supply (>15 l/min; 4 patients) was applied. **Supplementary Table 1** shows detailed individual clinical characteristics of all patients. 36 patients had COVID-19 comorbidities, among which hypertension (18 patients), smoking (10 patients) and obesity (9 patients) were the most common (all identified comorbidities are shown in **Supplementary Table 2**). For the presented study, patients were stratified based on the size of ground glass opacities (GGO, measured as percentage of lung parenchyma by CT) and type of the oxygen supply received. Patients were divided into 3 COVID-19 severity groups: mild (GGO ≤ 5, low-flow or without oxygen supply; n=16) moderate (GGO>5 and ≤20, low-flow oxygen supply; n=21) and severe (GGO≥21 or high-flow oxygen supply; n=22) (**Table 1** and **Supplementary Table 1**). Following COVID-19 recovery, peripheral blood was collected at two time regimes: up to 3 months (time-point I) and up to 6 months (time-point II) after the diagnosis, to investigate short- and long-lasting responses of the adaptive immune system in COVID-19 convalescents. None of the patients was either vaccinated against COVID-19 or re-infected with SARS-CoV-2 virus till the follow-up interview. This was followed by analysis of the most common post-COVID-19 syndrome symptoms, such as fatigue, dyspnea and cognitive dysfunctions, to check whether any of these symptoms might correlate with the observed immunological changes. The experimental pipeline of the study is presented on **Figure 1A**. The group of 13 age- and sex-matched individuals, without documented SARS-CoV-2 infection and no detectable IgG antibodies against SARS-CoV-2 S1/S2 in plasma, served as a healthy reference control group.

**Table 1:** Patients stratification into the COVID-19 severity groups: Healthy, Mild, Moderate and Severe.

COVID-19 severity group	Cumulative				Time-point I		Time-point II	
					14-90 days since diagnosis		91-180 days since diagnosis	
	n	Age	CT lung scan GGO [%]	Type of oxygen therapy	n	Age	n	Age
**HEALTHY**	13	47 (29-59)	NA	NA	NA	NA	NA	NA
**MILD**	16	45 (35-59)	2 (0-5)	-/low-flow passive	15	46 (35-59)	13	41 (35-53)
**MODERATE**	21	44 (27-63)	12 (6-20)	low-flow passive	20	44,5 (30-63)	12	43,5 (27-63)
**SEVERE**	22	46 (33-64)	34,5 (16-83)	passive/active	20	46 (33-64)	11	52 (36-64)

NA, not applicable.

All patients were hospitalized over the period of 25th June - 3rd November 2020 or 13th March – 23rd April 2021 at the Central Clinical Hospital of the Ministry of Interior and Administration in Warsaw. Selection is based on the size of ground glass opacities (GGO) measured as percentage of lung parenchyma by CT lung scan and type of the oxygen therapy: low-flow or without oxygen supply (mild), low-flow passive oxygen supply (moderate) and high-flow oxygen supply/active oxygen supply (severe). Age is shown as median with range in years. Cumulative data of all patients included into studies as well as data for patients analysed in each time-point (I and II) are shown.

Using plasma from convalescent patients and healthy donors, we examined the presence and temporal changes of the IgG antibodies fraction towards SARS-Cov-2 S1/S2 antigens. We have observed an increase in median IgG antibody levels that correlated with COVID-19 severity stages, with significant changes in moderate and severe post-COVID-19 patients (as compared to healthy and mild individuals). This was visible already at time-point I and further pronounced at the longer recovery time-point II (**Figure 1B**), also confirming the rationale behind our criteria for patients’ stratification.

### Cytokine Levels in Convalescent Patients’ Plasma

**Figure 2:**
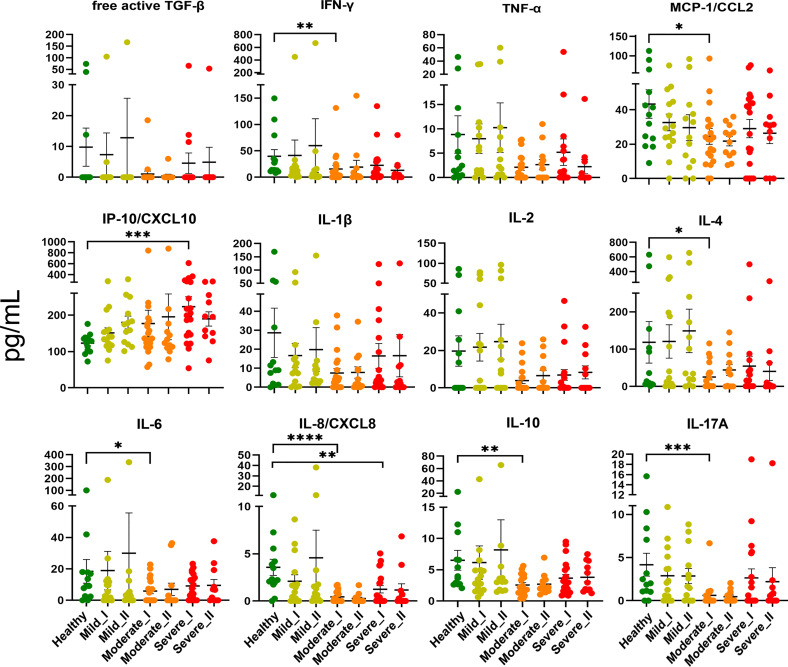
Plasma level of cytokines and chemokines from COVID-19 convalescents and controls. Quantification of cytokines and other mediators in plasma obtained from COVID-19 convalescents after severe (time-point I, n=20, time-point II, n=11), moderate (time-point I, n=20, time-point II, n=12) and mild disease (time-point I, n = 15, time-point II, n=13) at two time points after disease confirmation and from healthy controls (n = 13). Data represent individual values, mean (centre bar) ± SD (upper and lower bars). Statistical analysis by one-way ANOVA test, *p ≤ 0,05; **p ≤ 0,01; ***p ≤ 0,001; ****p ≤ 0,0001.

First, to assess whether the cytokine storm (one of the hallmarks of COVID-19 disease (25, 26) is still present in convalescent patients, we measured levels of 13 cytokines, chemokines and other immune mediators in plasma of convalescent patients and healthy controls (**Figure 2**). The cytokine storm has already been attenuated following recovery from COVID-19, as levels of TNF-α, free active TGF-β, IFN-γ, IL-1β, IL-2, IL-4, IL-6, IL-8, IL-10, IL-12p70, IL-17 and MCP-1/CCL2 during convalescence were not higher compared to healthy volunteers. However, the opposite effect, namely decreased levels of IL-4, IL-6, IL-8, IL-10 and IL-17, were observed in convalescent patients after moderate/severe COVID-19, compared to mild convalescents and healthy controls. Among all analyzed factors, chemokine IP-10/CXCL10, a marker of suppressed immune function and regulator of neutrophils function, was maintained at a higher level at time-point I in individuals convalescent from severe disease.

### Characterization and Dynamic Changes in CD4+ T Cell Subsets

**Figure 3:**
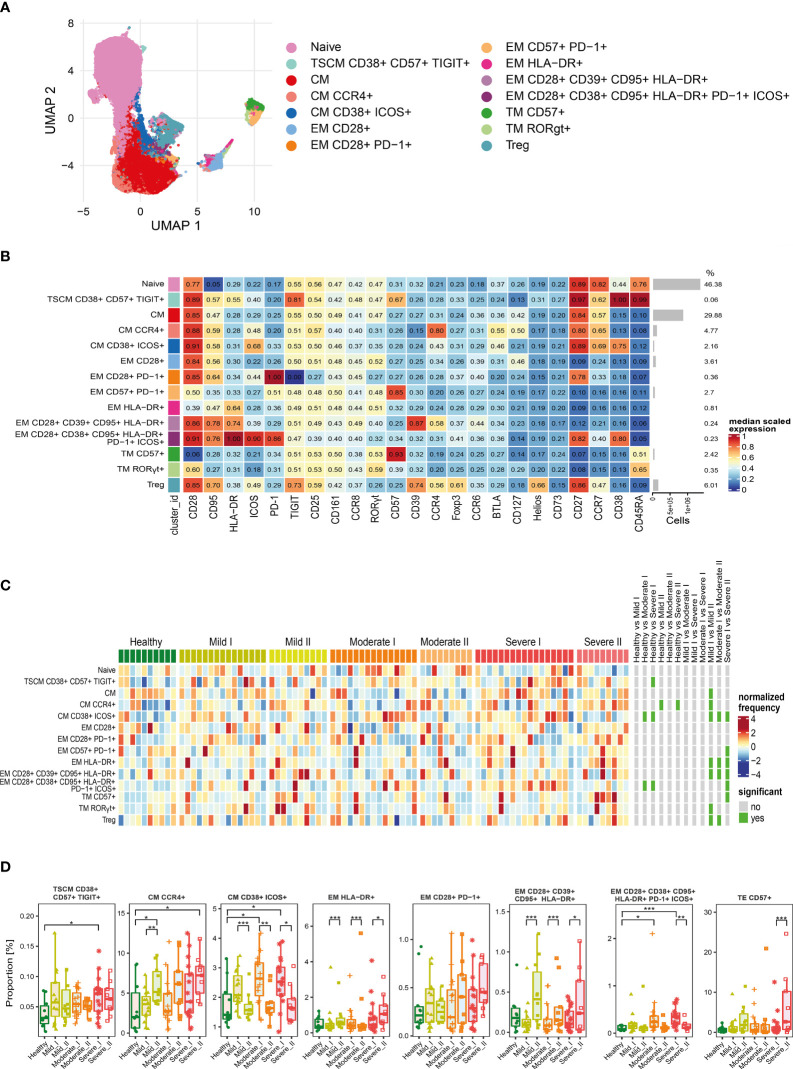
Unsupervised analysis of CD4+ T cells and their characterization. **(A)** Uniform Manifold Approximation and Projection (UMAP) UMAP representation of CD4+ T cell landscape. **(B)** Heat map representing different clusters identified by FlowSOM, with relative identity and percentages in healthy controls and convalescent patients. The color in the heat map represents the median of the arcsinh, 0–1 transformed marker expression calculated over cells from all the samples, varying from blue for lower expression to red for higher expression. Each cluster has a unique color assigned (bar on the left). Barplots along the rows (clusters) and values on the right indicate the relative sizes of clusters. **(C)** Differential analysis of all severity groups of COVID-19 convalescent patients (mild, moderate, severe), as well as healthy donors at the time points I and II. The heat represents arcsine-square-root transformed cell frequencies that were subsequently normalized per cluster (rows) to mean zero and standard deviation of one. The color of the heat varies from dark blue indicating relative under-representation to red indicating relative over-representation. Bars at the right indicate significantly differentially abundant clusters (green). **(D)** Differential proportion of selected clusters presented as % of CD4+ cells. *p ≤ 0,05; **p ≤ 0,01; ***p ≤ 0,001

**Figure 4:**
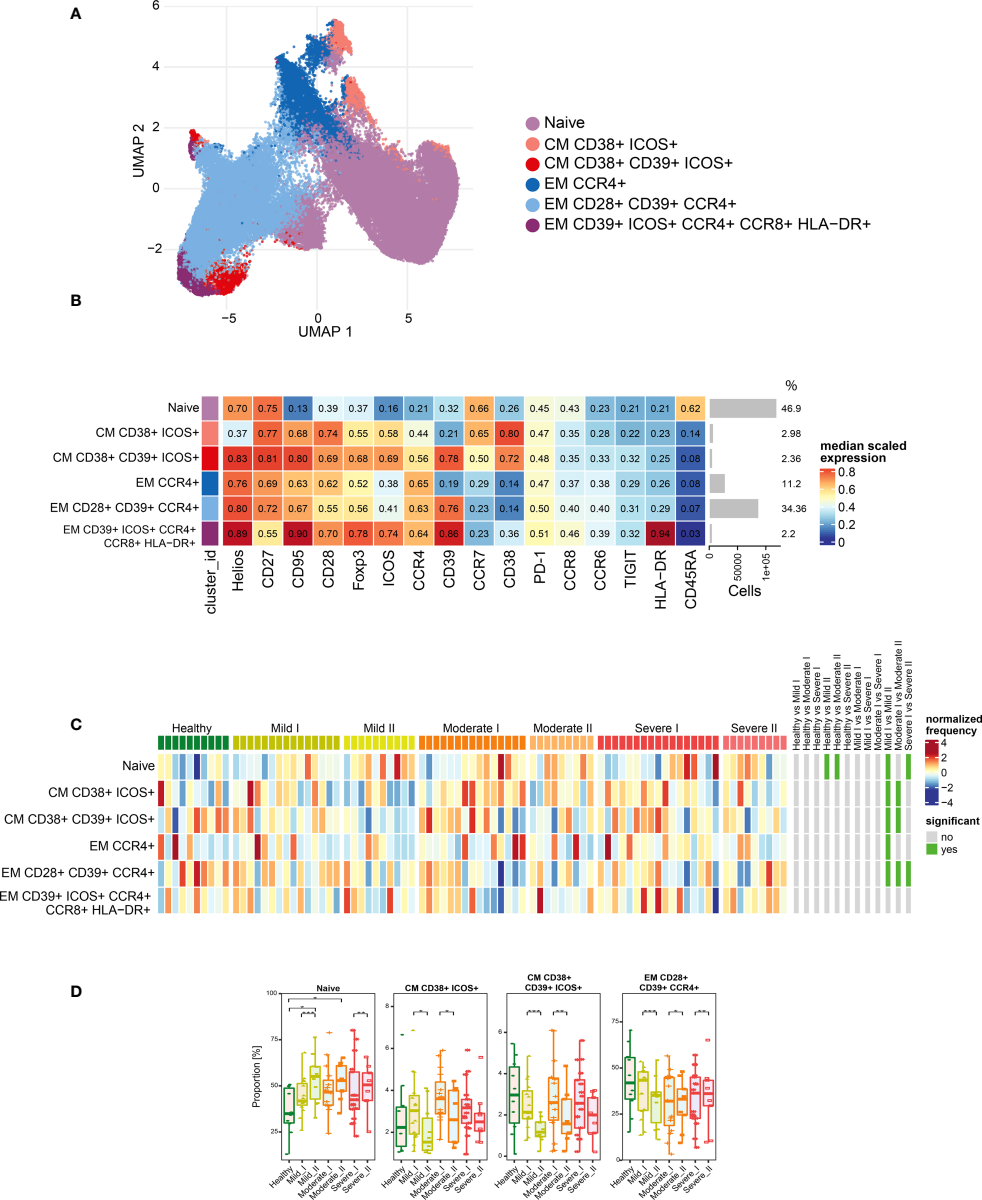
Unsupervised analysis of Treg cells and their characterization. **(A)** Uniform Manifold Approximation and Projection (UMAP) UMAP representation of Treg cell landscape. **(B)** Heat map representing different clusters identified by FlowSOM, with relative identity and percentages in healthy controls and convalescent patients. The color in the heat map represents the median of the arcsinh, 0–1 transformed marker expression calculated over cells from all the samples, varying from blue for lower expression to red for higher expression. Each cluster has a unique color assigned (bar on the left). Barplots along the rows (clusters) and values on the right indicate the relative sizes of clusters. **(C)** Differential analysis of all severity groups of COVID-19 convalescent patients (mild, moderate, severe), as well as healthy donors at the time points I and II. The heat represents arcsine-square-root transformed cell frequencies that were subsequently normalized per cluster (rows) to mean zero and standard deviation of one. The color of the heat varies from dark blue indicating relative under-representation to red indicating relative over-representation. Bars at the right indicate significantly differentially abundant clusters (green). **(D)** Differential proportion of selected clusters presented as % of Treg cells. *p ≤ 0,05; **p ≤ 0,01; ***p ≤ 0,001.

**Figure 5:**
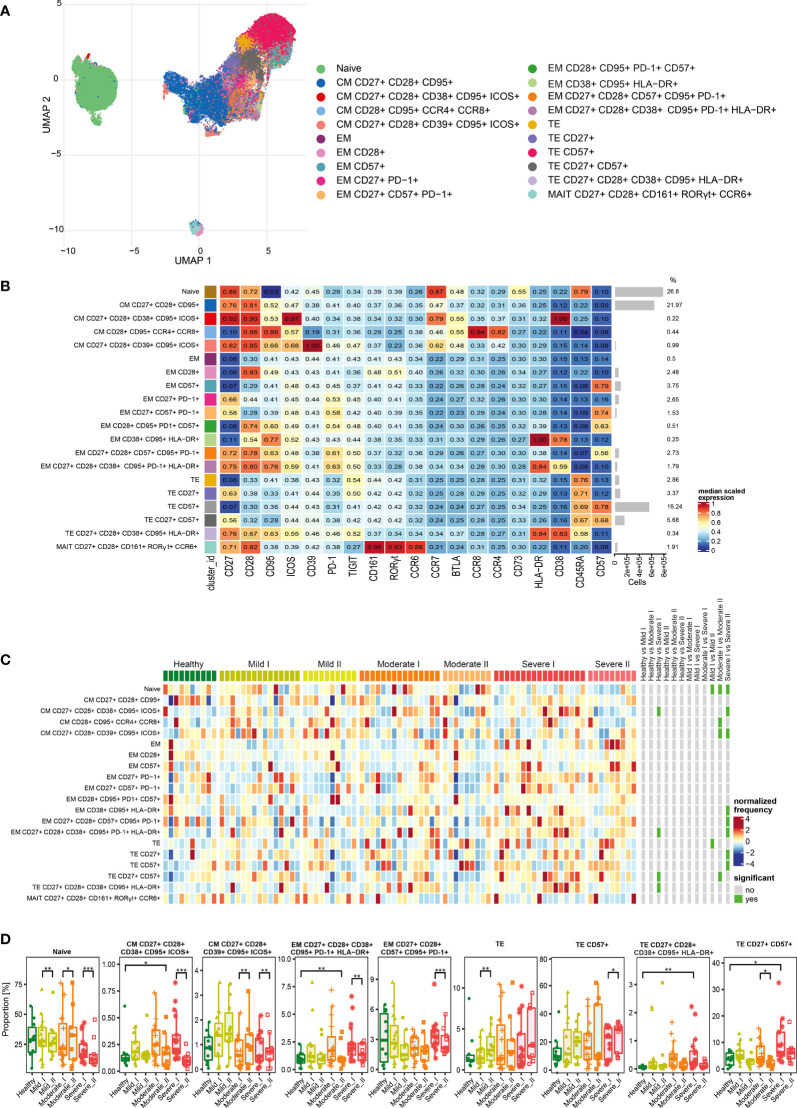
Unsupervised analysis of CD8+ T cells and their characterization. **(A)** Uniform Manifold Approximation and Projection (UMAP) UMAP representation of CD8+ T cell landscape. **(B)** Heat map representing different clusters identified by FlowSOM, with relative identity and percentages in healthy controls and convalescent patients. The color in the heat map represents the median of the arcsinh, 0–1 transformed marker expression calculated over cells from all the samples, varying from blue for lower expression to red for higher expression. Each cluster has a unique color assigned (bar on the left). Barplots along the rows (clusters) and values on the right indicate the relative sizes of clusters. **(C)** Differential analysis of all severity groups of COVID-19 convalescent patients (mild, moderate, severe), as well as healthy donors at the time points I and II. The heat represents arcsine-square-root transformed cell frequencies that were subsequently normalized per cluster (rows) to mean zero and standard deviation of one. The color of the heat varies from dark blue indicating relative under-representation to red indicating relative over-representation. Bars at the right indicate significantly differentially abundant clusters (green). **(D)** Differential proportion of selected clusters presented as % of CD8+ cells. *p ≤ 0,05; ** p ≤ 0,01; *** p ≤ 0,001

To assess immune system remodeling and T cell subset dynamic changes upon recovery from COVID-19 of different severity, we studied CD4+ T cells, regulatory T cells (Tregs) and CD8+ T cells in convalescent patients at time-points I and II, as well as in healthy donors, using 28 parameter full spectrum flow cytometry. Immune cell populations were first categorized into 2 major T cell lineages, based on identification of T lymphocytes as viable CD45+ CD3+ cells, and CD4 and CD8 markers (the full gating strategy is present on **Supplementary Figure 1**). Each cell type was further divided into subsets, based on functional marker expression, including activation and maturation status. Therefore, expression of markers to identify different subsets of naïve (CCR7+ CD45RA+ CD28+ CD27+), T stem cell memory cells (TSCM; CCR7+ CD45RA+ CD28+ CD27+ CD95+), central memory (CM; CCR7+ CD45RA- CD28+ CD27+), effector memory (EM; CCR7- CD45RA- CD28+/- CD27+/-), terminal effector (TE; CCR7- CD45RA+ CD28+/- CD27+/-), together with additional activation (CD38, HLA-DR, ICOS) and exhaustion/senescence (CD57, CD95, PD-1) or dysfunction (CD39) markers and finally differentiation subsets within Treg cells (identified as CD25hi CD127lo) have been assessed. Composition of basal markers for each functional subset are presented in **Table 2**. The general gating strategy is shown on **Supplementary Figure 1**. Unsupervised analysis was performed to investigate all potential changes at once and to visualize the complex responses (described in detail in Materials and Methods). The same approach was used to study CD4+, Treg and CD8+ T cells, and specific markers used for unsupervised analysis of each population are presented on **Figures 3B**, **4B**, **5B**, respectively.

**Table 2:** T cell phenotypes distinguished during manual and unsupervised analyses of CD4+ and CD8+ cells.

Cell population	Phenotype – specific marker composition
**Naïve**	CCR7+ CD45RA+ CD28+ CD27+
**TSCM – T stem cell memory**	CCR7+ CD45RA+ CD28+ CD27+ CD95+
**CM – central memory**	CCR7+ CD45RA- CD28+ CD27+
**EM – effector memory**	CCR7- CD45RA- CD28+/- CD27+/-
**TE – terminal effector**	CCR7- CD45RA+ CD28+/- CD27+/-
**Activated**	CD38+ HLA-DR+
**Exhausted**	CD57+ PD-1+
**Treg**	CD25+ CD127low
**Non suppressive/unstable Treg**	Foxp3low CD45RA-

For CD4+ cells, we generated 14 metaclusters, that represent different CD4+ T cell functional types, based on differential expression of activation, differentiation and exhaustion markers (all markers are presented on **Figure 3B**). The dimensionality reduction method UMAP was used to distinguish and visualize populations (**Figure 3A** and **Supplementary Figures 2–4**), which have been identified using FlowSOM metaclustering and which characteristics are presented as a heatmap (**Figure 3B**). Clusters were compared between healthy donors and all severity groups of COVID-19 convalescent patients (mild, moderate, severe) at time-points I and II by differential analysis (**Figures 3C, D**).

We found that the naïve CD4+ T cells, even though the most abundant, were similar between all groups and times (**Figures 3B, C**). Central memory (CM) T cells clusters were highly represented (**Figure 3B**) and significantly changed among CD4+ cells, although two different schemes of responses were observed (**Figure 3D**). We noticed that the frequency of CM cells expressing CCR4 increased dependently on the severity at time-point I and further increased at time-point II, exhibiting significant expansion in severe individuals (**Figure 3D**), whereas activated CM population expressing CD38 and ICOS, even if significantly increased after moderate and severe COVID-19 at 3 months after COVID-19 (time-point I), strongly decreased at longer recovery till 6 months after COVID-19 (time-point II) in all groups (**Figure 3D**). Pools of activated effector memory (EM) cells generally increased at time-point II in a severity-dependent manner. These included activated EM cells with solely expression of HLA-DR, which showed high abundance in severe convalescents at time-point II, and EM cells co-expressing CD28, CD39 and CD95, together with HLA-DR (**Figure 3D**). Cluster of the effector memory cells expressing exhaustion marker PD-1 showed a similar increasing trend (**Figure 3D**). Moreover, also the terminal effector memory cells (TE) that express CD57, marker associated with exhaustion/senescence, significantly increased in severe convalescents at longer recovery time-point II (**Figure 3D**). On the other hand, EM cells expressing CD38, CD95, HLA-DR, PD-1 and ICOS showed the dynamics similar to CM cells expressing CD38 and ICOS, namely increase at time-point I followed by significant drop at time-point II (**Figure 3D**). We have also found a very small population of CD4+ T stem cell memory cells (TSCM) with expression of CD38, CD57, and TIGIT, that increased at time I in severe convalescent patients, though this was not further observed after longer recovery at time II (**Figure 3D**). Altogether these data indicate that the long-term recovery of severe convalescents leads to decrease in number of activated central memory CD4+ cells, increase in population of CCR4+ CM cells which indirectly support immunosuppressive activity of Treg cells, accompanied by skewed polarization of CD4+ T cells towards the exhaustion/senescent state.

As our panel contained CD25, CD127 and transcription factors Foxp3 and Helios, we were able to identify a population of regulatory T cells. Then, following analysis of functional markers by FlowSOM metaclustering (all markers are presented on **Figure 4B**), we identified and visualized 6 subsets of Treg cells (**Figures 4A, B** and **Supplementary Figures 2, 5, 6**). Among Treg cells, we observed an increase in the most abundant subset of naïve cells at both time-points of recovery (**Figures 4B–D**). This was accompanied by a significant decrease in CD38 and ICOS-expressing CM Treg cells at time-point II (**Figures 4C, D**), similarly to the changes described above in CD4+ cells (**Figure 3D**). However, in Treg cells such an effect was especially visible during recovery in mild and moderate convalescents, as compared to the severe group (**Figure 4D**). Effector Tregs that express CD28, CD39 and CCR4, established a highly represented subset (**Figures 4B–D**). They showed time-dependent significant decrease in mild convalescents or increase observed in moderate and severe (**Figure 4D**). Altogether, these data suggest either a block in Treg maturation, or an increase in influx of naïve Treg cells, correlating with reduced population of potentially immunosuppressive Tregs in mild and moderate, but not in severe convalescents upon long-term recovery.

### Characterization and Dynamic Changes in CD8+ T Cell Subsets

After analysis of functional markers in CD8+ T cells subset (all markers are shown on **Figure 5B**), followed by FlowSOM meta-clustering, we have identified 20 functional clusters, visualized by UMAP (**Figure 5A** and **Supplementary Figures 2, 7, 8**) and presented as a heat map (**Figure 5B**). Then, as described above, differential analysis has been performed to compare all severity groups and the analyzed time-points.

The naïve population was also the most abundant among CD8+ cells (**Figures 5B–D**). However, we noticed the time-dependent decrease of naïve cells number, especially visible in severe convalescents (**Figure 5D**). Central memory cells were also highly represented among CD8+ population (**Figure 5B**). Among those, central memory cells expressing CD27, CD28, CD39, CD95 and ICOS showed increased frequencies especially in mild convalescents, and decrease in moderate and severe. On the other hand, CM cells expressing CD38, CD95 and ICOS showed reduced frequencies visible at time-point II, coming back to the baseline level of healthy donors (**Figures 5C, D**). Identified clusters of effector memory (EM) cells were either not frequent or not significantly changed (**Figure 5D**). The dynamics towards increased senescence/exhaustion was clearly visible among terminal effector cells of severe convalescents at time-point II. The frequency of TE CD8+ cells expressing CD57 has generally increased among convalescent patients and represented around 40% of all CD8+ cells at time-point II (**Figures 5B–D**). Identified terminal effector CD8+ cells expressing activation markers HLA-DR and CD38 (CD27+ CD28+ CD38+ CD95+ HLA-DR+) constituted a small population increased at time-point I in the severe group, although it reversed to the level of healthy donors at the second time-point (**Figure 5D**). In general, at time-point II we noticed largely reduced populations of CM, EM and TE CD8+ cells expressing CD27 and CD28 markers (**Figure 5D**), indicating attenuation of CD8+ T cells activation, expansion and differentiation from memory into effector CD8+ T cells (35). Finally, we identified a cluster of mucosal associated invariant T (MAIT) cells, however the frequencies did not differ either between severity types or time-points (**Figures 5B, C**).

Altogether we found that the long-term post-COVID-19 recovery leads to the time-dependent decrease of naïve and central memory populations of CD8+ T cells, accompanied by significant increase in terminal effector subsets expressing the exhaustion/senescence markers, observed in severe convalescents. This could suggest that CD8+ cells exhibit strong polarization towards an exhausted state visible in severe post-COVID-19 patients upon long-term recovery.

### *Ex Vivo* Production of Cytokines

Next, we have studied production of seven different functional molecules in CD4+, Treg and CD8+ T cells, including IFN-γ, IL-17, IL-2, TNF-α, TGF-β and granzyme-B, along with expression of degranulation marker CD107a (LAMP-1). First, the classical approach, based on two-dimensional cell type identification by manual sequential gating, has been performed. In each group of identified cells (CD4+, CD4+Tregs, CD8+), the levels of above-mentioned cytokines have been measured using the full spectrum Cytek Aurora cytometer. The full gating strategy is presented on **Supplementary Figure 9**.

The total production of each cytokine is summarized in **Supplementary Figure 10**. We found that CD4+ cells from severe convalescent patients had significantly higher capacity to produce TNF-α, IL-2 and CD107a at time-point I, compared to healthy controls and mild/moderate COVID-19 convalescents (**Supplementary Figure 10A**). Increased production of these cytokines was also observed for Foxp3+ Treg cells (**Supplementary Figure 10B**). Importantly, Tregs from severe convalescents also showed increased production of IL-17, IL-2+IL1-7 and IFN-γ at time-point I. This was observed neither for mild or moderate patients nor for healthy donors. However, at time-point II, we noticed a significant decrease in the production of all above mentioned cytokines, in both CD4+ and Treg cells, especially in severe convalescent patients (**Supplementary Figures 10A, B**). CD8+ T cells did not exhibit significantly higher production of most of the analyzed cytokines. Only in severe convalescents at time-point II, we observed slight decrease in production of CD107a and visible, though not statistically significant, increased production of Granzyme B (**Supplementary Figure 10C**).

**Figure 6:**
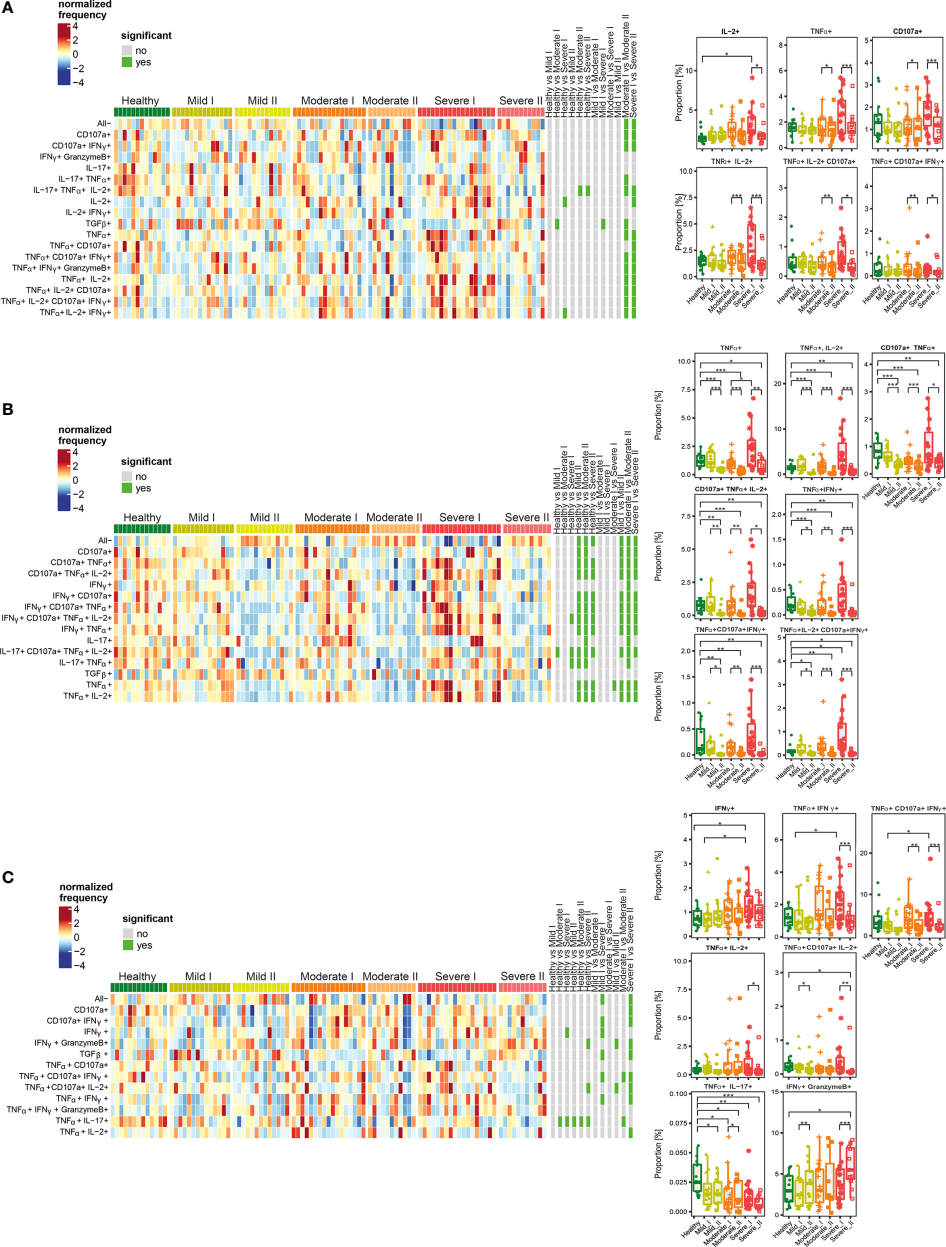
Unsupervised analysis of cytokine production by CD4+ **(A)**, Treg **(B)** and CD8+ **(C)** cells from convalescent patients after mild, moderate and severe COVID-19 as well as healthy donors analyzed at time I and time II. Upper panels - Heatmaps showing cell clusters identified by FlowSOM with differential analysis of all severity groups of COVID-19 convalescent patients (mild, moderate, severe) as well as healthy controls at the time points I and II. The heat represents arcsine-square-root transformed cell frequencies that were subsequently normalized per cluster (rows) to mean of zero and standard deviation of one. The color of the heat varies from dark blue indicating relative under-representation to red indicating relative over-representation. Bars at the right indicate signi ficant differentially abundant clusters (green). The population without any activity (negative for all markers) has been marked as “All-”. Lower panels – differential proportion of selected clusters presented as % of analyzed cells. *p ≤ 0,05; **p ≤ 0,01; ***p ≤ 0,001.

We then investigated CD4+, Treg and CD8+ T cells polyfunctionality by analyzing simultaneous production of TNF-α, CD107a, IFN-γ, IL-2, IL-17, granzyme-B and TGF-β by mild, moderate and severe COVID-19 convalescent patients at recovery time-point I and time-point II, as well as by healthy donors (**Figure 6**). Polyfunctional studies, considering several cytokines produced simultaneously by T cells, have enabled deeper deciphering of T cell functions during COVID-19 convalescence. Based on the use of seven functional markers for FlowSOM metaclustering, we could discriminate up to 22 different populations of CD8+, CD4+ and Treg cells able to produce one or more molecules simultaneously, in different combinations. Next, the differential analysis together with statistical analysis has been performed for each cluster and shown on a heatmap (**Figures 6A–C**, upper panels), as well as graphs showing differential proportions (presented for selected clusters) (**Figures 6A–C**, lower panels).

Among CD4+ T cells, we identified clusters of cells characterized by higher simultaneous production of different combinations of IL-2, TNF-α, CD107a and IFN-γ, at the recovery time-point I in severe convalescent patients, compared to moderate and mild post-COVID19, as well as healthy individuals (**Figure 6A**). The observed upregulation in simultaneous production of those cytokines in identified polyfunctional clusters persisted up to 3 months after recovery (time-point I), and was followed by significant decrease at time-point II, especially in severe patients (**Figure 6A**).

Functional clusters identified among Treg cells showed high proportion of cells with simultaneous production of different combinations of TNF-α, IL-2, CD107a, and IFN-γ (which have been found before in the entire population of CD4+ cells) at time-point I in severe post-COVID-19 patients (**Figure 6B**). However, similarly to CD4+ cells, later during recovery at time-point II, the amount of cells among almost all identified clusters significantly decreased and dropped even below the level found in healthy donors. Interestingly, such a significant drop in cytokines production was visible not only in severe post-COVID patients, but also in moderate and mild, even though previous increase observed at time I was not as strong as in severe cases.

Among CD8+ T cells (**Figure 6C**), the responses and polyfunctionality changes were not as strong as in CD4+ and Treg cells. We identified clusters characterized by higher proportion of IFN-γ-, TNF-α- and CD107a-producing cells in severe convalescents at time-point I. As in other T cell populations, such increased production of cytokines at time-point I was followed by decrease at time-point II. Additionally, a strong, severity- and time-dependent decrease in production of TNF-α together with IL-17 was observed. Specifically for CD8+ cells, a population showing simultaneous production of IFN-γ together with Granzyme B was identified. Production of these cytokines increased dependent on disease severity and time, showing significant increase in severe convalescents at time-point II, compared to healthy individuals. Such an effect was predominant in severe patients at time II.

Altogether, these data show that CD4+, Tregs, as well as CD8+ cells from severe convalescent individuals, at time-point I presented functional types which predominantly produced proinflammatory TNF-α, IL-2, IFN-γ and CD107a at high levels. Longer recovery (time-point II) led to strong decrease of most of the analyzed factors, not only in severe, but also in moderate patients. This has often exhibited levels lower than in healthy donors. Finally, the increased proportion of CD8+ cells producing Granzyme B together with IFN-γ was specifically characteristic for severe post-COVID-19 patients at time II, indicating unresolved inflammation. Such a trend was also found in CD4+ cells, although without statistical significance.

### Post-Acute COVID-19 Syndrome in Convalescent Patients After Mild, Moderate and Severe COVID-19

The appearance of post-acute COVID-19 syndrome (PACS, signs and symptoms beyond 12 weeks), also called “long COVID”, has often been observed, significantly decreasing the quality of patients’ life (16). These symptoms include tiredness, fatigue (extreme tiredness), shortness of breath and dyspnea but also a broad range of cognitive symptoms such as difficulties in concentration and memory (“brain fog”), sleep problems, depression and others (36, 37). Importantly, it seems that they appear not only in convalescents recovering from severe COVID-19, but also in those who suffered from moderate, mild and even asymptomatic forms of disease. Even if the appearance of long COVID is already broadly recognized, the molecular mechanisms leading to those long-term effects are still not clear. Nevertheless, the long-lasting immune system-related factors, T cell exhaustion and neuroinflammation are proposed as very probable drivers (38). Therefore we have assessed the common symptoms of the prolonged PACS in the convalescent patients, depending on the disease severity. The data have been collected at the 1-year follow-up interview made by a clinician, in which patients have been asked about presence of cognitive symptoms/brain fog, fatigue and dyspnea/shortness of breath, all lasting over 3 months.

**Supplementary Table 4** shows that long COVID, independently on the type of PACS symptoms, was found in 81% (13/16) of severe, 70% (12/17) of moderate and 61% (8/13) of mild convalescents, therefore indicating that PACS is severity-independent. Moreover we observed that different types of symptoms showed different distributions and co-existence.

Looking closer into the PACS symptoms we found that dyspnea or fatigue lasting over 3 months, presented the most prevalent PACS symptoms observed in 50% of patients after severe COVID-19, accompanied by cognitive PACS symptoms found in 43,7% of cases (**Table 3**). In the majority of severe convalescents, co-existence of at least two symptoms was observed (**Supplementary Table 4**). In moderate or mild convalescents, dyspnea was present in a much lower number of cases and represented 17,6% or 15%, respectively. Interestingly, prolonged cognitive impairment was predominant and reported by 70% of moderate convalescent patients, accompanied with fatigue reported by 47% and dyspnea appearing only in 17,6% (**Table 3**). Such cognitive impairment reported by moderate convalescents often co-existed with fatigue but not dyspnea symptoms. Moreover, fatigue never existed without additional cognitive dysfunction (**Supplementary Table 4**). Finally, a significant number of mild convalescents still indicated PACS, which has been represented by fatigue (38% of cases) and cognitive dysfunctions (30% of cases), but not dyspnea (only 15% of cases) (**Table 3**). The clear co-existence of any symptoms was not observed in the mild group, however the presence of cognitive dysfunctions together with fatigue was still visible in 2 out of 8 mild cases with PACS (**Supplementary Table 4**). In none of the patients, stroke or heart attack appeared after COVID-19.

**Table 3:** Appearance of post- acute COVID-19 syndrome (PACS, signs and symptoms beyond 12 weeks) in post-COVID-19 patients from all severity groups.

COVID-19 severity group	Cognitive dysfunction	Fatigue	Dyspnea
**MILD** (n=13)	30,7% (4/13)	38,4% (5/13)	15% (2/13)
**MODERATE** (n=17)	70,5% (12/17)	47,1% (8/17)	17,6% (3/17)
**SEVERE** (n=16)	43,7% (7/16)	50,5% (8/16)	50,0% (8/16)

Data have been collected during an interview made by a clinician.

Altogether we found that among the PACS symptoms, fatigue and cognitive dysfunctions were highly represented in all groups, including mild post-COVID-19 patients, often co-existed, and had no significant relationship with the severity of the disease. On the other hand, dyspnea dominated in convalescents recovering from severe forms of the disease. Its presence was much lower in moderate or mild post-COVID-19. Interestingly, prolonged cognitive dysfunctions were predominant in patients after moderate COVID-19, and significantly present not only in severe, but also in patients recovering from mild COVID-19.

## Discussion

Persistent symptoms of post-acute COVID-syndrome - PACS (named also “long COVID”), representing incomplete recovery from COVID-19, lead to continued health-related problems, reported by over 30% of all convalescents (regardless of the severity of the initial infection) (2, 10, 39, 40). As they can lead to significant complications, such health conditions represent an emerging medical and global problem in the nearest future (10). Therefore, studies which analyze convalescent subjects months after the disease of different severity are crucial to address and develop an integrative healthcare approach.

Most investigations describe PACS about 1-3 month after recovery, or just report severe convalescents, and scanty data exist that compare changes in T cell subsets from convalescent individuals who had experienced mild, moderate or severe COVID-19. Here we show several immune parameters collected at two time-points, i.e., up to 3 and 6 months after mild, moderate or severe infection, and provide information about the persistence of PACS. We have found that recovery from severe COVID-19 leads to functional shift after the first 3 months post-infection, resulting in polarization towards an exhausted/senescent state of CD4+ and CD8+ T cells and perturbations in CD4+ Treg subsets, clearly visible at 6 months after COVID-19. In terms of functionality, these changes are accompanied by attenuation of the unstable Th1/Th17-like Treg phenotype and proinflammatory polarization of CD4+ (which was still observed at 3 months after primary infection). CD8+ cells which were particularly polarized towards the terminal effector state, simultaneously increased proportion of Granzyme B and IFN-γ-producing cells, indicating prolonged cytotoxic capabilities and unresolved pro-inflammatory traits.

On the other hand, mild convalescents do not show polarization of T cells towards the exhausted/senescent state but are characterized by increased population of naïve Tregs, concurrently with decrease in CM and EM Tregs. Furthermore, we have discovered that several post-acute COVID symptoms, such as fatigue and cognitive dysfunction are severity-independent and are not necessarily related to exhausted/senescent state and dysfunctions of CD4+, CD8+ and Treg cells found in severe convalescents.

Immune dysregulation has been associated with post-acute COVID-19 recovery (41). Several previous reports showed that T cell activation/exhaustion remain elevated following SARS-CoV-2 infection (14, 32, 33) as well as in severe convalescents (42). Particularly, T cell exhaustion seems to play a significant role in post-COVID-19, as blockade of the PD-1/PD-L1 axis led to restoration of T cell function and reversal of the observed post-acute COVID-19 immune abnormalities (43). Consistently with this, we also identified increased presence of exhausted (PD-1-expressing) and senescent (CD57-expressing) EM and TE CD4+ and CD8+ T cells in severe convalescents. Such phenotyping remodeling has been noticed during COVID-19 infection (33), nevertheless, we showed that significant skewing towards the exhausted/senescent phenotype is observed after the first 3 months of recovery in severe patients.

We identified CD8+ T cells as particularly polarized towards the exhausted/senescent state in severe, but not mild and moderate convalescents after 6 months-long recovery. The frequency of terminal effector CD8+ T cells expressing CD57, finally achieved about 40% of all CD8+ T cells. Different subsets of CD8+ cells additionally exhibited a decrease of CD27 and CD28 expression, characteristic for attenuation of T cell activation and expansion as well as age-associated decline of immune function (44, 45). Therefore, prevalence of subsets with increased CD57 expression, together with decreased expression of CD27 and CD28, indicates expansion of CD8+ T cells polarized towards a dysfunctional terminally differentiated state, lacking full effector capability. Such population can play a significant role in various diseases or conditions, associated with chronic immune activation such as, among others, HIV infection, cancer, intracellular infections, chronic pulmonary diseases and autoimmune diseases, as well as has a great influence on age-related changes in the immune system (46, 47). This was observed together with a strong decrease in naïve CD8+ T cell numbers. Our data are in agreement with reports in which elevated exhaustion of CD8+ cells has been proposed as an indicator for progression and prognosis of COVID-19 disease (48), as well as lower frequency of naïve CD8+T cells has been identified in the short-term post-COVID-19 studies (49). Therefore, such a highly represented dysfunctional exhausted/senescent CD8+ population might be involved in the post-COVID pathogenesis but also will provide the suppressive tumor-promoting microenvironment (41). Altogether this indicates that convalescents recovering from severe COVID-19 might face the long-lasting dysfunctions in T cell responses, that result in potentially weaker immunity to other infections, cancer and other diseases, which can significantly affect health or medical treatment of patients.

The number of Tregs is significantly reduced in COVID-19 patients and perturbances in Treg activity correlate with COVID-19 severity, risk of respiratory failure and higher mortality (50, 51). We saw that different subsets of Treg show different dynamics between the short (until 3 months) and the long-term (until 6 months) post-COVID-19 responses. This included a decreased population of ICOS-expressing CM Treg cells, which was most abundant in mild convalescents at the second time-point. ICOS is highly expressed by activated CD4+ and Treg cells, and improves the survival, proliferation, also favouring the suppressive function of Treg cells (52). Our observations are in accordance with cytometric and transcriptomic analyses, which revealed a distinct Treg signature in severe patients, resembling immunosuppressive and tumor infiltrating features and correlating with poor prognosis (50). Simultaneously, long-term data revealed significant increase in Treg naïve subset in mild and moderate, but not severe, convalescents. Overall, these results could indicate a lower suppressive functions and either a block in maturation, or an increased influx of naïve Treg cells into the circulation in convalescents, especially at longer time after mild or moderate COVID-19. Moreover, at the first time-point Tregs from severe convalescents produce more IL-2, IL-2 plus IL-17 and IFN-γ, suggesting the presence of polarization into unstable Th1/Th17-like states. These data imply improper function of Treg cells during COVID-19 recovery, possibly affecting incomplete resolution of inflammation.

Concerning T cell polyfunctionality, CD4+, Treg, as well as CD8+ T cells from moderate and severe convalescents presented functional types which simultaneously produced high levels of Th1 and Th17 cytokines, (as revealed by intracellular staining showing different combinations of TNF-α, IL-2, CD107a and IFN-γ), until 3 months of recovery. This is consistent with other studies which indicated proinflammatory Th1/Th17 state of T cells after severe COVID-19 (26, 33, 53). These data suggest impaired/slowed resolution of inflammation, which is otherwise crucial to prevent the progression from non-resolving acute inflammation to persistent chronic inflammation (54). An effective resolution of inflammation is crucial for severe convalescents. We found that longer recovery (3-6 months) leads to strong inhibition of simultaneous production of those cytokines, and additionally TNF-α plus IL-17, often below the level observed in healthy controls. Our finding is supported by other studies, which showed decreased levels of Th1-polarized CD4+ cells during recovery from COVID-19 (55).

Severe post-COVID-19 patients exhibited an increased proportion of CD8+ cells producing Granzyme B together with IFN-γ upon long recovery, suggesting the persistence of a strong cytotoxic T cell responses. This is consistent with data showing prolonged enhancement of Granzyme B production by cytotoxic T lymphocytes in post-recovery patients (56). Also, IFN-γ produced by CD8+ T cells, which remained persistently high at 8 months after infection, has been recently considered a long COVID predictor (57). This could imply that CD8^+^CD57^+^ T cells exhibiting enhanced cytotoxic potencies, and impaired proliferative capability, are recruited to the peripheral blood and the unresolved chronic inflammatory state is maintained in severe convalescents long after recovery from COVID-19. These observations might provide future opportunities for prevention and treatment. Blocking/inhibition of Granzyme B production is currently investigated in different therapies, including autoimmune diseases (58, 59). Also, development of pro-resolution strategies and implementation of so-called “resolution pharmacology” was proposed (60) to prevent the prolonged chronic inflammatory state, leading to a plethora of diseases (54). Such therapeutic modalities might be also an opportunity for severe COVID-19 convalescent individuals to prevent the post-acute COVID syndrome and further possible complications.

We found that long-COVID-19 effects, such as fatigue and/or cognitive dysfunction, appear significantly in all convalescents, often showing co-existence. The incidence of the post-COVID syndrome is commonly estimated at 10-35%, while for previously hospitalized patients it may reach 85% (61). Thus, the role of different variants of SARS-CoV-2 remains to be elucidated.

Pathogenesis of PACS is complex, and dysregulated T cell functions have been proposed as one of its drivers (62, 63). Even with limitations of our studies, such as relatively basic clinical review of PACS symptoms and relatively low number of cases in each group (about 20-25), together with a broad range of age (without dividing into age-related groups) of only male patients, our data suggest that PACS symptoms are present also in mild/moderate convalescents and are not exclusively related to exhaustion/senescence of CD4+ and CD8+ T cells, or to a defective resolution of inflammatory phenomena. Such observation is still preliminary, based on the limited number of cases and addresses only T cells subsets, however indicates that different mechanisms, which might depend or not on COVID-19 severity, can be involved in different types of PACS symptoms. Similar conclusions have been driven by the recently released comprehensive sc-RNASeq studies of patients until 6 months post-infection (17). Authors showed that even mild convalescents might have long-lasting changes in the transcriptome of blood cells. Our observation is also consistent with deep multi-omic studies reporting that patients with mild and severe/acute COVID-19 exhibited similar PACS symptoms, and different immune endotypes correlated with different PACS symptoms (18). This suggests that different repertoire of immune cells might be responsible for different manifestations of post-COVID symptoms. However, those studies have focused on a shorter period after severe COVID-19 infection, whereas our data showed that there is a further significant remodeling of the immune response at longer post-COVID time (at 3-6 months).

We can conclude that dysregulated immunity of CD4+, CD8+ and Treg cells is, to an extent, maintained for the first 3 months post COVID-19, followed by a functional switch partially visible at 3-6 month. The magnitude of observed phenomena is different for severe and mild/moderate patients. This manifests itself as long-lasting attenuation of T cell activation and expansion, and skewing towards an exhausted/senescent state, particularly in CD8+ T cells. Together with prolonged unresolved inflammation, it might result in dysfunctions in T cell responses causing a potentially weaker immunity. These severe immune dysfunctions specifically co-existed together with the pulmonary complications/dyspnea, however this was not found for PACS symptoms such as fatigue and cognitive dysfunction, which were present not only in severe, but also in a substantial percentage of mild and moderate convalescents. On the other hand, the long-term responses in mild or moderate COVID-19 convalescents result in lower polarization towards exhausted/senescent state, attenuation of the immunosuppressive subsets, associated with increased amount of circulating naïve Treg cells. Therefore this data, even if performed on a limited number of cases, indicate that some PACS problems, such as fatigue and cognitive dysfunction are rather severity-independent. We propose that they are probably not related to the polarization of T cells towards exhaustion/senescence state, which has been found by us predominantly in severe convalescents, however this needs further deeper studies.

Finally, whilst it is a growing area of medicine and research, we chose not to study individuals who received recently developed anti-COVID-19 vaccines. We assume that this would make investigation of the mechanisms more complex, possibly leading to potentially skewed interpretation and conclusions. Moreover, with a big probability all analyzed patients were infected with the alpha variant of SARS-CoV-2 virus, as identification of the first cases of delta variant in Poland began in June 2021, so after we have finalized our studies. Future deep, long-lasting studies of COVID-19 of all severity stages, together with selected age-related and male/female groups are needed to better understand the nature of association of the long-term defects in the immune system with prolonged responses that include metabolic changes of immune cells, as well as emerging neurological manifestations. Such studies will potentially uncover the relation of dysregulated immunity, acute or chronic immune responses and different PACS symptoms. In any case, either the search for long COVID predictors or any treatment to prevent the post-COVID syndrome and further possible complications is mandatory in all patients with SARS-CoV-2 infection, and not only in those suffering from severe/acute COVID-19.

## Data Availability Statement

The original contributions presented in the study are included in the article/**Supplementary Material**. Further inquiries can be directed to the corresponding author.

## Ethics Statement

The studies involving human participants were reviewed and approved by ethics committee of the Central Clinical Hospital of the Internal Affairs and Administration Ministry in Warsaw (Decision No 151/2020) with informed consent of enrolled individuals. The study was performed in accordance with the latest version of the Declaration of Helsinki and the guidelines for good clinical practice. The patients/participants provided their written informed consent to participate in this study.

## Author Contributions

MW, MB-O, and PC performed experiments; MW, PC, DS, and JS performed data analyses; PC and KP coordinated cooperation with clinics; MH, AM, MD, and WW coordinated and performed patients identification and clinical analysis; MH, AM, and PC organized and coordinated convalescents blood donation and questionnaire; KS performed CT lung analysis; MW, PC, JS, and KP conceptualized experiments; KP conceptualized and supervised the project; MW, PC, DS, and KP prepared figures and tables; MW, PC, JS, DS, and KP prepared draft of the manuscript; AC, SDB, JS, MW, and PC were involved in data discussion and interpretation; MW, PC, DS, JS, MH, SDB, AC, and KP prepared and reviewed final version of the manuscript; All authors have read and agreed to the published version of the manuscript.

## Funding

This work has been funded by grant from Foundation for Polish Science TEAM-TECH Core Facility Plus/2017–2/2 (POIR.04.04.00-00-23C2/17-00) co-financed by the European Union under the European Regional Development Fund to KP. JS was supported by EMBO Scientific Exchange Grant (Short-Term Fellowship) STF-8905.

## Conflict of Interest

The authors declare that the research was conducted in the absence of any commercial or financial relationships that could be construed as a potential conflict of interest.

## Publisher’s Note

All claims expressed in this article are solely those of the authors and do not necessarily represent those of their affiliated organizations, or those of the publisher, the editors and the reviewers. Any product that may be evaluated in this article, or claim that may be made by its manufacturer, is not guaranteed or endorsed by the publisher.
