# Supplementary material for: Remodeling of T Cell Dynamics During Long COVID Is Dependent on Severity of SARS-CoV-2 Infection
Source: Front Immunol. 2022 Jun 10;13:886431. doi: 10.3389/fimmu.2022.886431 (PMC9226563; doi:10.3389/fimmu.2022.886431)
Supplement: Supplementary file 1 [file DataSheet_1.pdf]

Supplementary Information

Wiech M. et al., Remodeling of T cell dynamics during long COVID is dependent on severity of SARS-CoV-2 infection

Supplementary Table 1

The individual clinical characteristics of all patients.

Patient ID	Age	COVID-19 severity	CT lung scan - GGO [%]	COVID-19-related therepy			Days from diagnosis to convalescence	Blood colection time-point I (14-90 days since diagnosis)			Blood colection time-point II (91-180 days since diagnosis)		
				Oxygen supply	Steroids	Others		Days from diagnosis to blood collection	Anti-SARS-CoV-2 S1/S2 IgG [BAU/ml]	Anti-SARS-CoV-2 IgM [AU/ml]	Days from diagnosis to blood collection	Anti-SARS-CoV-2 S1/S2 IgG [BAU/ml]	Anti-SARS-CoV-2 IgM [AU/ml]
1	64	SEVERE	74	High-flow active	-	-	28	-	-	-	102	600,6	8,1
2	49	SEVERE	22	Low-flow passive	Dexametasone	-	17	83	>1040	2,7	-	-	-
10	44	SEVERE	50	Low-flow passive	Dexametasone	Convalescent plasma transfer	14	49	655,2	4,7	132	1872	0,9
12	52	SEVERE	40	Low-flow passive	Dexametasone	Convalescent plasma transfer	19	54	712,4	17,9	135	756,6	4,6
21	61	SEVERE	34	Low-flow passive	Dexametasone	-	11	68	416	1,7	157	384,8	0,3
23	50	SEVERE	26	Low-flow passive	Dexametasone	Convalescent plasma transfer	15	52	824,2	4,4	140	1853,8	0,8
27	46	SEVERE	28	Low-flow passive	Dexametasone	Remdesivir	7	24	299	1,8	-	-	-
31	64	SEVERE	30	High-flow passive	Dexametasone	-	17	54	728	5,7	136	1908,4	1,3
32	62	SEVERE	36	Low-flow passive	Dexametasone	Convalescent plasma transfer	7	43	639,6	3,6	126	793	0,5
37	36	SEVERE	16	High-flow passive	Dexametasone	Remdesivir	24	-	-	-	112	374,4	4
50	49	SEVERE	83	High-flow active	-	-	14	29	>1040	21,8	98	>2080	2
51	46	SEVERE	26	Low-flow passive	Dexametasone	-	23	46	962	37,5	115	1560	22,9
54	52	SEVERE	33	Low-flow passive	-	-	18	38	522,6	6,5	95	>2080	1,2
55	51	SEVERE	24	Low-flow passive	Dexametasone	-	12	23	873,6	12,6	-	-	-
64	43	SEVERE	35	Low-flow passive	Dexametasone	-	17	45	>2080	5,1	-	-	-
65	43	SEVERE	30	Low-flow passive	Dexametasone	-	16	25	>2080	29,6	-	-	-
66	33	SEVERE	45	Low-flow passive	Dexametasone	-	15	28	>2080	11,9	-	-	-
69	44	SEVERE	30	Low-flow passive	Dexametasone	-	9	18	887	30,3	-	-	-
73	44	SEVERE	50	Low-flow passive	Dexametasone	-	14	28	>2080	4,3	-	-	-
74	37	SEVERE	60	Low-flow passive	Dexametasone	-	20	46	1590	4,2	-	-	-
75	44	SEVERE	35	Low-flow passive	Dexametasone	-	8	20	1340	30,7	-	-	-
77	34	SEVERE	70	Low-flow passive	Dexametasone	-	12	26	2080	13,2	-	-	-
3	42	MODERATE	12	Low-flow passive	Dexametasone	-	27	82	473,2	0,2	180	595,4	0,1
11	42	MODERATE	18	Low-flow passive	Dexametasone	-	7	43	481	5,4	126	1682,2	0,8
15	63	MODERATE	19	Low-flow passive	-	-	8	53	332,8	13,8	144	855,4	3,4
17	37	MODERATE	12	Low-flow passive	Dexametasone	-	15	44	301,6	2,1	137	429	0,6
18	45	MODERATE	10	Low-flow passive	-	-	14	53	322,4	6,3	143	358,8	1,8
19	52	MODERATE	17	Low-flow passive	-	Convalescent plasma transfer	15	63	488,8	2,4	152	767	0,5
20	58	MODERATE	10	Low-flow passive	Dexametasone	-	16	74	8,58	1,3	169	652,6	0,9
28	60	MODERATE	7	Low-flow passive	-	-	11	74	520	4,1	164	756,6	0,8
33	61	MODERATE	7	Low-flow passive	-	-	10	69	527,8	1	153	886,6	0,3
56	30	MODERATE	16	Low-flow passive	-	-	3	30	481	11,2	91	1749,8	1,3
57	56	MODERATE	6	Low-flow passive	Dexametasone	-	9	23	551,2	7,8	-	-	-
58	58	MODERATE	17	Low-flow passive	Dexametasone	-	7	26	442	1,7	-	-	-
59	40	MODERATE	8	Low-flow passive	Dexametasone	-	7	41	224,12	2,8	98	213,2	0,7
62	27	MODERATE	15	Low-flow passive	Dexametasone	Convalescent plasma transfer	14	-	-	-	116	1112,8	3,3
63	41	MODERATE	15	-	-	-	7	20	>2080	22,6	-	-	-
67	44	MODERATE	8	-	-	-	13	42	>2080	5,2	-	-	-
68	42	MODERATE	10	Low-flow passive	Dexametasone	-	10	14	>2080	65,6	-	-	-
71	42	MODERATE	15	Low-flow passive	Dexametasone	-	13	40	1400	42,8	-	-	-
72	56	MODERATE	15	Low-flow passive	Dexametasone	-	15	31	>2080	4,2	-	-	-
76	52	MODERATE	10	Low-flow passive	Dexametasone	Remdesivir	9	30	>2080	2,6	-	-	-
78	38	MODERATE	20	-	Dexametasone	-	10	44	1240	7,4	-	-	-
4	52	MILD	2	-	Dexametasone	-	19	77	126,62	22,1	173	288,6	10
5	47	MILD	1	Low-flow passive	-	-	20	75	190,84	2,2	179	265,2	1,3
6	35	MILD	0	Low-flow passive	-	-	11	67	127,14	2,7	165	236,86	0,7
7	35	MILD	2	-	-	-	21	56	69,42	5,7	152	97,5	1,7
8	40	MILD	0	-	-	-	9	55	47,06	0,3	138	53,82	0,2
9	41	MILD	3	-	-	-	14	58	69,94	39,2	143	67,86	9,1
13	53	MILD	3	Low-flow passive	-	-	17	57	293,8	2,6	148	340,6	0,5
14	46	MILD	2	Low-flow passive	Dexametasone	-	12	67	167,18	2,3	158	205,92	1,6
16	51	MILD	1	-	Dexametasone	-	18	66	434,2	16,5	-	-	-
22	48	MILD	3	Low-flow passive	Dexametasone	-	12	58	293,8	4,3	149	231,14	0,7
24	38	MILD	2	-	-	-	7	43	42,12	4,5	132	86,32	1,3
26	47	MILD	2	Low-flow passive	-	-	11	41	551,2	2,4	96	1118	0,3
52	36	MILD	0	-	Dexametasone	Remdesivir	17	77	145,6	0,4	147	88,4	0,4
53	40	MILD	0	-	-	-	13	-	-	-	108	134,42	0,8
70	43	MILD	5	-	-	-	5	17	820	6,5	-	-	-
79	59	MILD	2	Low-flow passive	Dexametasone	-	12	34	>2080	5,2	-	-	-
25	29	HEALTHY	NA	NA	NA	NA	NA	NA	<9,8	0,1	NA	NA	NA
34	58	HEALTHY	NA	NA	NA	NA	NA	NA	<9,8	0,1	NA	NA	NA
39	51	HEALTHY	NA	NA	NA	NA	NA	NA	<9,8	0,1	NA	NA	NA
40	54	HEALTHY	NA	NA	NA	NA	NA	NA	<9,8	0,1	NA	NA	NA
41	47	HEALTHY	NA	NA	NA	NA	NA	NA	<9,8	0,1	NA	NA	NA
42	50	HEALTHY	NA	NA	NA	NA	NA	NA	<9,8	0,2	NA	NA	NA
43	56	HEALTHY	NA	NA	NA	NA	NA	NA	<9,8	0	NA	NA	NA
44	59	HEALTHY	NA	NA	NA	NA	NA	NA	<9,8	0,2	NA	NA	NA
46	47	HEALTHY	NA	NA	NA	NA	NA	NA	<9,8	0,4	NA	NA	NA
47	35	HEALTHY	NA	NA	NA	NA	NA	NA	<9,8	0,1	NA	NA	NA
48	37	HEALTHY	NA	NA	NA	NA	NA	NA	<9,8	0,1	NA	NA	NA
49	34	HEALTHY	NA	NA	NA	NA	NA	NA	<9,8	0,1	NA	NA	NA
61	43	HEALTHY	NA	NA	NA	NA	NA	NA	<9,8	0,1	NA	NA	NA

NA - not applicable

Supplementary Table 2

Comorbidities identified in COVID-19 convalescent patients.

		Comorbidities in patients with COVID-19										
Patient ID	COVID-19 severity	Asthma	Pulmonary embolism	Diabetes mellitus	Obesity	Hypertension	Cardiovascular disease	Chronic kidney disease	Chronic liver disease	Cancer	Neurological disorders	Smoking
1	SEVERE	No	No	No	No	Yes	Yes	No	No	No	No	No
2	SEVERE	No	No	No	Yes	No	Yes	No	No	No	No	Yes
10	SEVERE	No	No	Yes	No	No	No	No	No	No	No	No
12	SEVERE	No	No	No	No	Yes	No	No	No	No	No	Yes
21	SEVERE	No	No	No	No	Yes	No	No	No	No	No	No
23	SEVERE	No	No	No	No	No	No	No	No	No	No	No
27	SEVERE	No	No	No	Yes	No	No	No	No	No	No	No
31	SEVERE	No	No	Yes	No	Yes	Yes	No	Yes	No	No	No
32	SEVERE	Yes	No	Yes	Yes	No	No	No	Yes	No	No	Yes
37	SEVERE	No	No	No	No	No	No	No	No	No	No	No
50	SEVERE	No	No	No	No	No	No	No	No	No	No	No
51	SEVERE	No	No	No	Yes	No	No	No	No	No	No	No
54	SEVERE	No	No	No	No	No	No	No	No	No	No	No
55	SEVERE	No	No	No	Yes	No	No	No	No	No	Yes	No
64	SEVERE	No	No	Yes	Yes	Yes	No	No	No	No	No	No
65	SEVERE	No	No	No	No	No	No	No	No	No	No	No
66	SEVERE	No	No	No	No	No	No	No	No	No	No	No
69	SEVERE	No	No	No	No	No	No	No	No	No	No	No
73	SEVERE	No	No	No	No	No	No	No	No	No	No	Yes
74	SEVERE	No	No	No	No	Yes	No	No	No	No	No	No
75	SEVERE	No	No	No	No	No	No	Yes	No	No	No	No
77	SEVERE	No	No	No	No	No	No	No	Yes	No	No	No
3	MODERATE	No	No	No	No	No	No	No	No	No	Yes	No
11	MODERATE	No	No	No	No	No	No	No	No	No	No	No
15	MODERATE	No	No	Yes	No	Yes	No	No	No	No	No	No
17	MODERATE	No	No	No	No	No	No	No	No	No	No	No
18	MODERATE	No	No	No	No	No	No	No	No	No	No	No
19	MODERATE	No	No	No	No	Yes	No	No	No	No	No	No
20	MODERATE	No	No	No	No	Yes	No	No	No	No	No	No
28	MODERATE	No	No	Yes	No	No	No	No	No	No	No	No
33	MODERATE	No	No	No	No	No	No	No	No	No	No	Yes
56	MODERATE	No	No	No	No	No	No	No	No	No	No	No
57	MODERATE	No	Yes	No	No	Yes	No	No	No	No	Yes	No
58	MODERATE	No	No	No	No	Yes	Yes	No	No	No	Yes	No
59	MODERATE	No	No	No	No	No	No	No	No	No	No	No
62	MODERATE	No	No	No	No	No	No	No	No	No	No	Yes
63	MODERATE	No	No	No	No	No	No	No	No	No	No	No
67	MODERATE	No	No	No	No	No	No	No	No	No	No	No
68	MODERATE	No	No	No	No	Yes	No	No	No	No	No	No
71	MODERATE	No	No	No	No	No	No	No	No	No	No	No
72	MODERATE	No	No	No	No	Yes	No	No	No	No	No	No
76	MODERATE	No	No	No	Yes	Yes	Yes	No	No	No	No	No
78	MODERATE	No	No	No	No	No	No	No	No	No	No	No
4	MILD	No	No	No	No	No	Yes	No	No	No	Yes	No
5	MILD	No	No	No	No	No	No	No	No	No	No	Yes
6	MILD	No	No	No	No	No	No	No	No	No	No	Yes
7	MILD	No	No	No	No	No	No	No	No	No	No	No
8	MILD	No	No	No	No	No	No	No	No	No	No	No
9	MILD	No	No	No	No	Yes	No	No	No	No	No	No
13	MILD	No	No	No	No	No	No	No	No	No	No	No
14	MILD	No	No	No	No	No	No	No	No	No	No	No
16	MILD	No	No	No	Yes	Yes	No	No	No	No	No	No
22	MILD	No	No	No	Yes	Yes	No	No	No	No	No	No
24	MILD	No	No	No	No	No	No	No	No	No	No	Yes
26	MILD	No	No	No	No	No	No	No	No	No	No	No
52	MILD	No	No	No	No	No	No	No	No	No	No	No
53	MILD	No	No	No	No	No	No	No	No	No	No	No
70	MILD	No	No	No	No	No	No	No	No	No	No	Yes
79	MILD	No	No	No	No	Yes	No	No	No	No	Yes	No

Supplementary Table 3
Antibodies used.

Specificity	Fluorochrome	Clone	Manufacturer	Cat No	Panel	Titer used [ul]
CD3	BUV496	UCHT-1	BD	612940	T cell functionality	1,25
CD4	Alexa Fluor-700	RPA-T4	Biolegend	300526	T cell functionality	0,5
CD8	APC-Cy7	RPA-T8	Biolegend	301016	T cell functionality	0,6
Foxp3	PE	PCH101	Invitrogen	12-4776-42	T cell functionality	0,6
IL-2	APC	MQ1-17H12	Biolegend	500310	T cell functionality	0,6
IL-17	PE-Cy7	BL168	Biolegend	512315	T cell functionality	0,6
Granzyme B	PerCP-Cy5.5	QA18A28	Biolegend	396412	T cell functionality	2,5
CD107a	Alexa Fluor-488	H4A3	BD	567006	T cell functionality	1
TGF-β	BV421	TW4-9E7	BD	562962	T cell functionality	5
TNF-α	BV605	MAb11	Biolegend	502936	T cell functionality	0,6
IFN-γ	BV510	B27	Biolegend	506540	T cell functionality	2,5
CD45	Krome Orange	J33	Beckman Coulter	B36294	Phenotyping	1,25
CD3	APC-AF750	UCHT1	Beckman Coulter	A94680	Phenotyping	0,625
CD4	cFluor 584	SK3	Cytek	R7-20041	Phenotyping	0,625
CD8	BV510	RPA-T8	Biolegend	301048	Phenotyping	0,625
CD127	APC R700	HIL-7R-M21	BD	565185	Phenotyping	1,25
CD25	BV421	2A3	BD	564033	Phenotyping	0,625
CD45RA	BUV395	HI100	BD	740298	Phenotyping	0,313
CCR7	BV785	G043H7	Biolegend	353230	Phenotyping	5
CD27	PECy7	O323	Biolegend	302838	Phenotyping	1,25
CD28	BUV737	CD28.2	BD	612815	Phenotyping	1,25
CD38	APCFire810	HIT2	Biolegend	303550	Phenotyping	5
CD57	Pacific Blue	HNK-1	Biolegend	359608	Phenotyping	0,625
HLA-DR	BUV805	G46-6	BD	748338	Phenotyping	1,25
CD95	PECy5	DX2	Biolegend	305610	Phenotyping	0,625
PD1	BV650	EH12	BD	564104	Phenotyping	5
Foxp3	APC	PCH101	Invitrogen	17-4776-42	Phenotyping	2,5
Helios	FITC	22F6	Invitrogen	11-9883-82	Phenotyping	0,125
RORgt	PE	Q21-559	BD	563081	Phenotyping	5
CCR6	BV711	G034E3	Biolegend	353436	Phenotyping	2,5
CCR4	BB700	1G1	BD	566475	Phenotyping	2,5
CD161	PerCP	HP-3G10	Biolegend	339934	Phenotyping	5
CD73	BUV496	AD2	BD	750061	Phenotyping	1,25
ICOS	BUV563	DX29	BD	741421	Phenotyping	2,5
BTLA	BUV661	J168+540	BD	750250	Phenotyping	2,5
CCR8	PE Dazzle 594	433H	BD	566888	Phenotyping	1,25
CD39	PECy5.5	BA54	Beckman Coulter	B55385	Phenotyping	5
TIGIT	BV605	A15153G	Biolegend	372712	Phenotyping	5

Supplementary Table 4

The individual appearance of post-acute COVID syndrome (PACS) symptoms. Data have been collected during an interview made by a clinician.

Patient ID	COVID-19 severity	Cognitive symptoms	Fatigue	Dyspnea
2	SEVERE	-	+	+
10	SEVERE	-	-	-
12	SEVERE	-	+	+
21	SEVERE	+	-	+
27	SEVERE	-	-	-
31	SEVERE	-	-	-
32	SEVERE	+	-	+
37	SEVERE	+	+	+
51	SEVERE	+	-	+
64	SEVERE	+	+	-
65	SEVERE	-	-	+
66	SEVERE	-	+	-
69	SEVERE	+	-	+
74	SEVERE	-	+	-
75	SEVERE	+	+	-
77	SEVERE	-	+	-
3	MODERATE	+	+	-
11	MODERATE	+	+	-
15	MODERATE	-	-	-
17	MODERATE	+	+	-
18	MODERATE	+	+	+
19	MODERATE	-	-	-
20	MODERATE	-	-	-
28	MODERATE	-	-	-
33	MODERATE	+	+	-
56	MODERATE	+	+	-
57	MODERATE	+	-	+
58	MODERATE	+	-	-
62	MODERATE	+	+	-
63	MODERATE	+	-	+
67	MODERATE	+	+	-
71	MODERATE	+	-	-
72	MODERATE	-	-	-
4	MILD	-	-	-
5	MILD	+	+	-
6	MILD	-	+	-
7	MILD	+	+	-
8	MILD	-	-	-
13	MILD	-	-	-
14	MILD	-	-	-
22	MILD	-	+	-
24	MILD	-	-	+
26	MILD	+	-	-
53	MILD	+	-	-
70	MILD	-	-	-
79	MILD	-	+	+

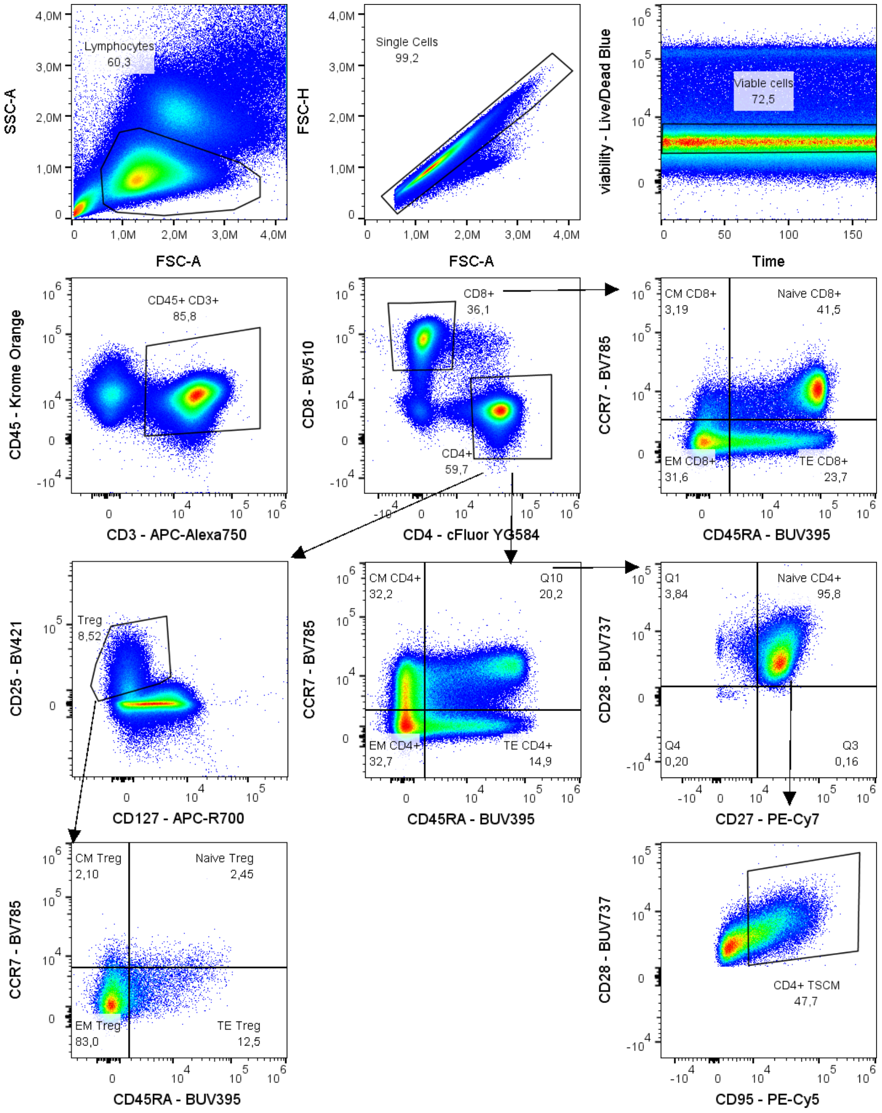

Supplementary Figure 1

The gating strategy that was used to identify populations of CD4+ and CD8+ T cells as well as Treg cells followed by identification of naive, central memory, effector memory and terminal effector as well as TSCM cells.

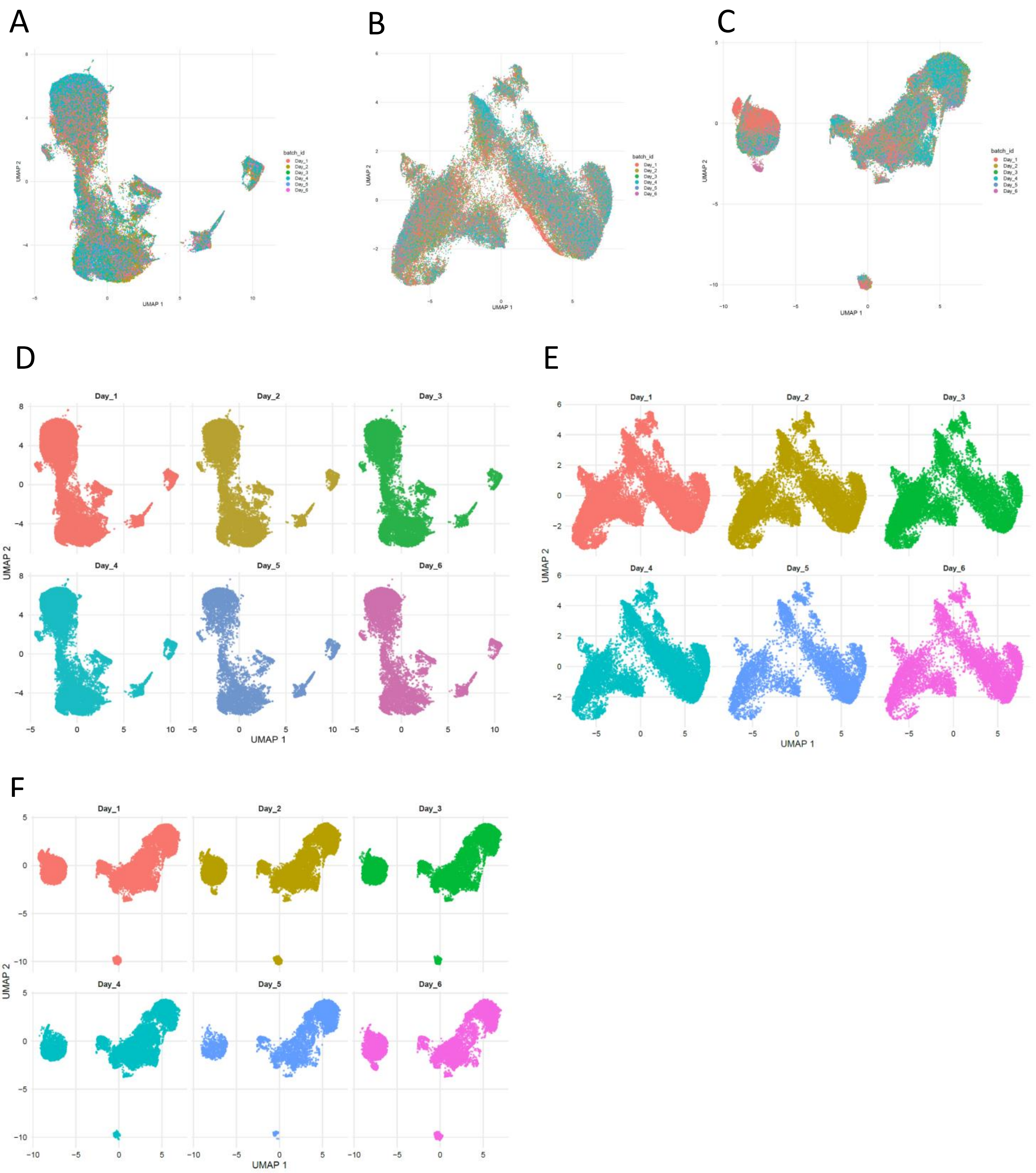

Supplementary Figure 2

A-C. UMAP graph overlaid for multiple batches (referred to different acquisition days – Day_1-6) for CD4+ T cells (A), CD4+Treg cells (B), CD8+ T cells (C). D-F. Projection of UMAP graph stratified by batch; each color indicates a different day for CD4+ T cells (D), CD4+Treg cells (E) and CD8+ T cells (F).

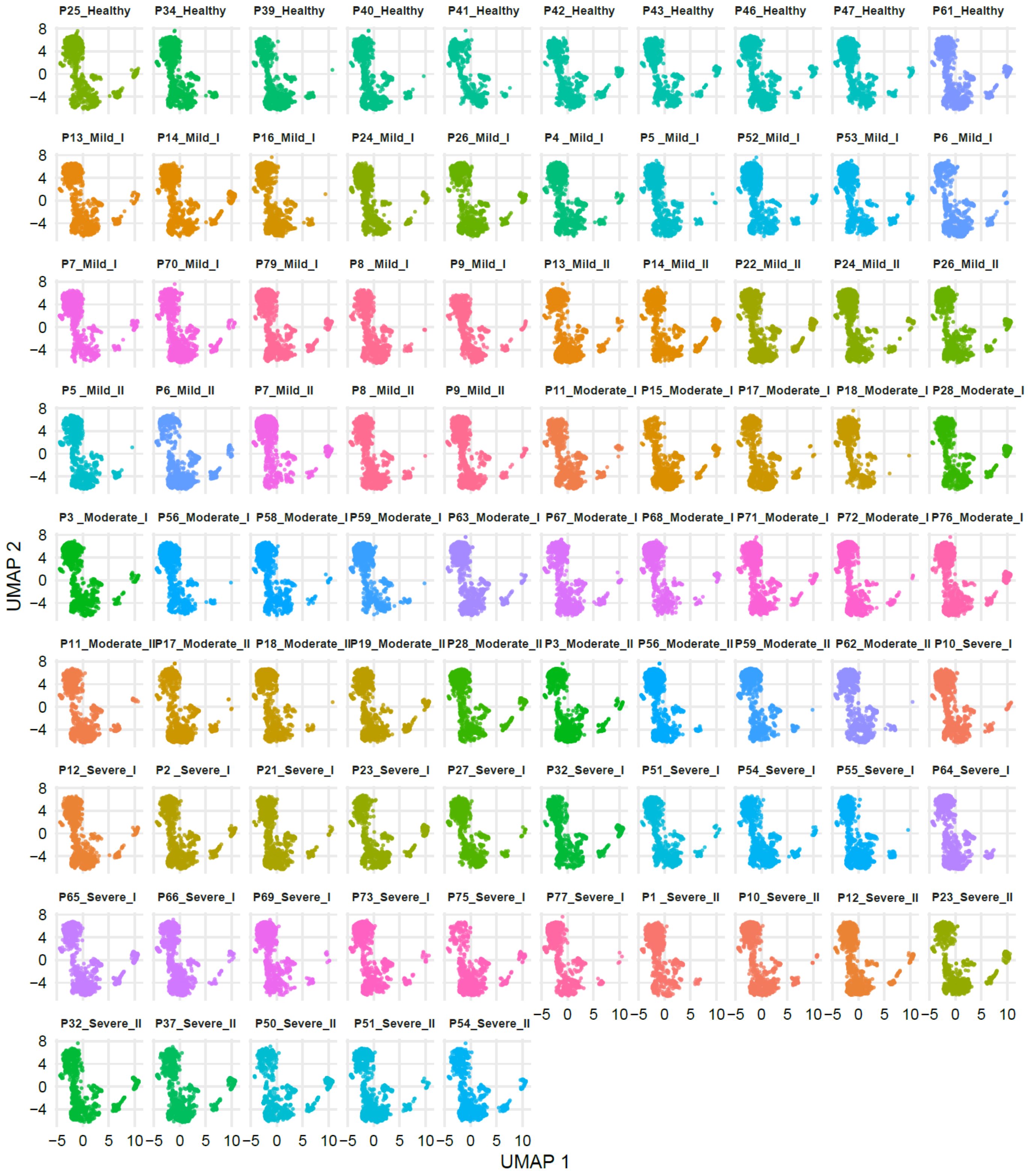

Supplementary Figure 3

CD4+ UMAP graphs stratified by patient sample: patient (P) number, Healthy control, COVID-19 severity (Mild, Moderate, Severe), and time-point I or II are given.

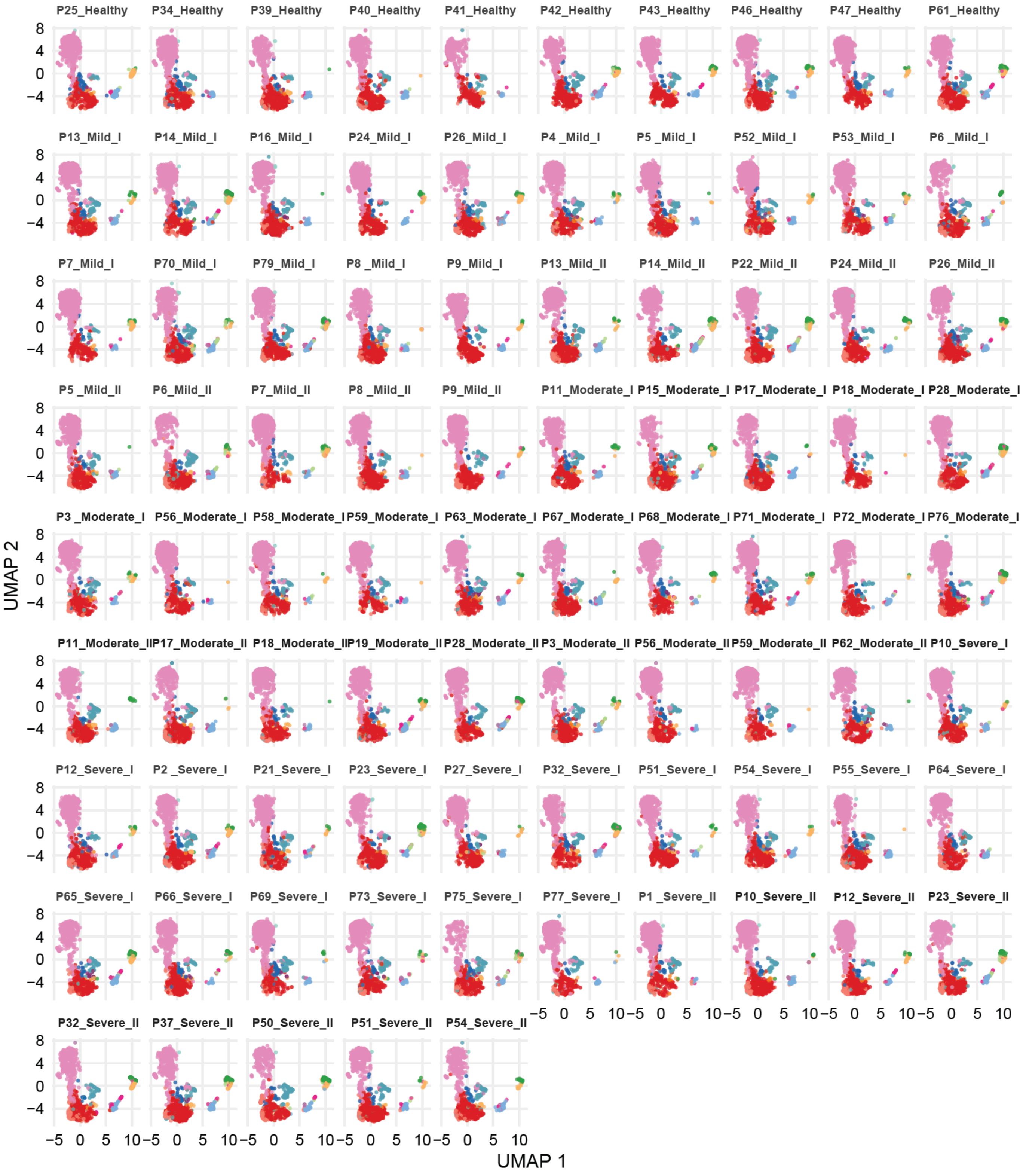

Supplementary Figure 4

Projection of CD4+ UMAP graphs stratified by patient sample showing the FlowSOM clusters: : patient (P) number, Healthy control, COVID-19 severity (Mild, Moderate, Severe), and time-point I or II are given

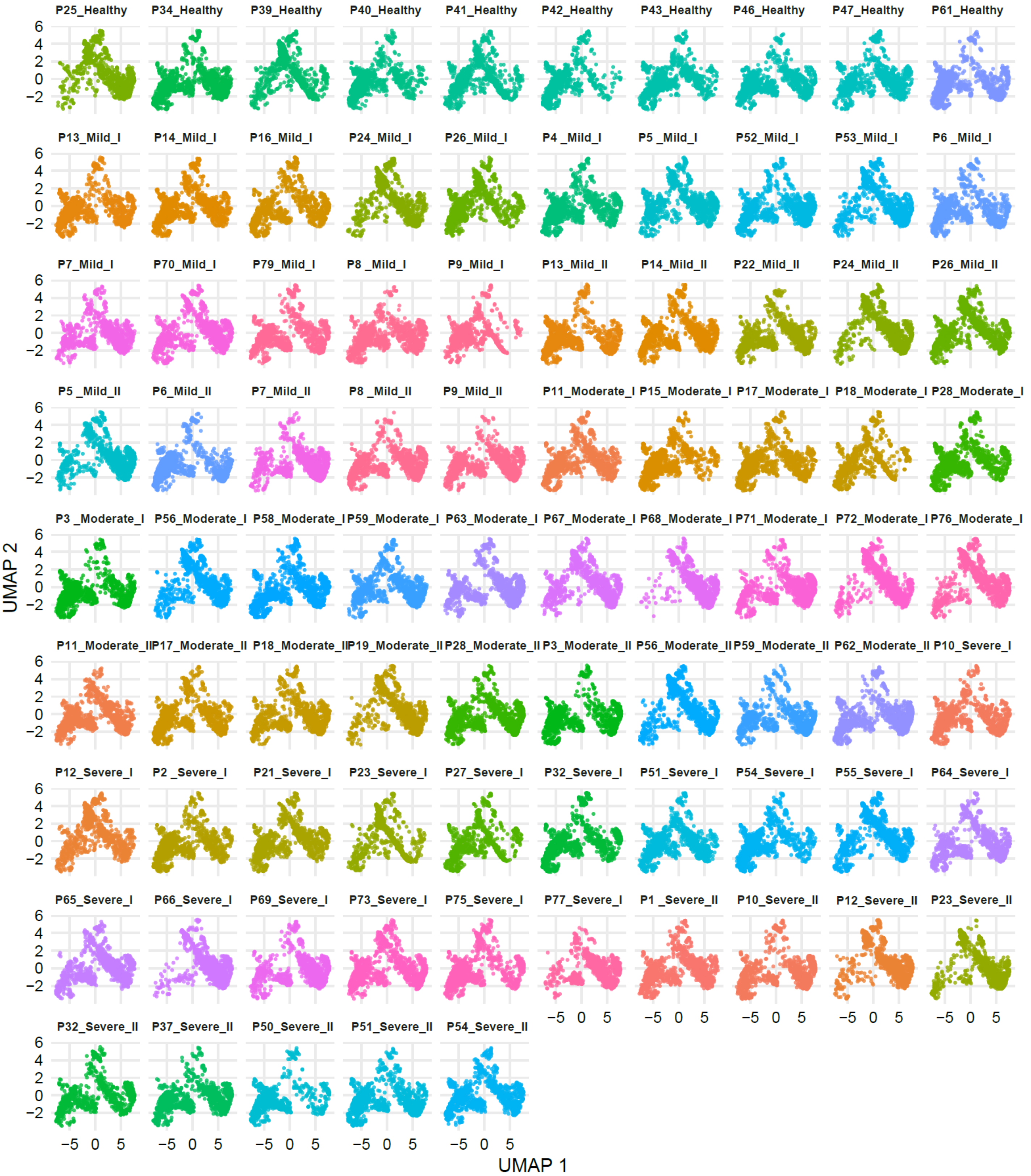

Supplementary Figure 5
CD4+Treg UMAP graphs stratified by patient sample: patient (P) number, Healthy control, COVID-19 severity (Mild, Moderate, Severe), and time-point I or II are given.

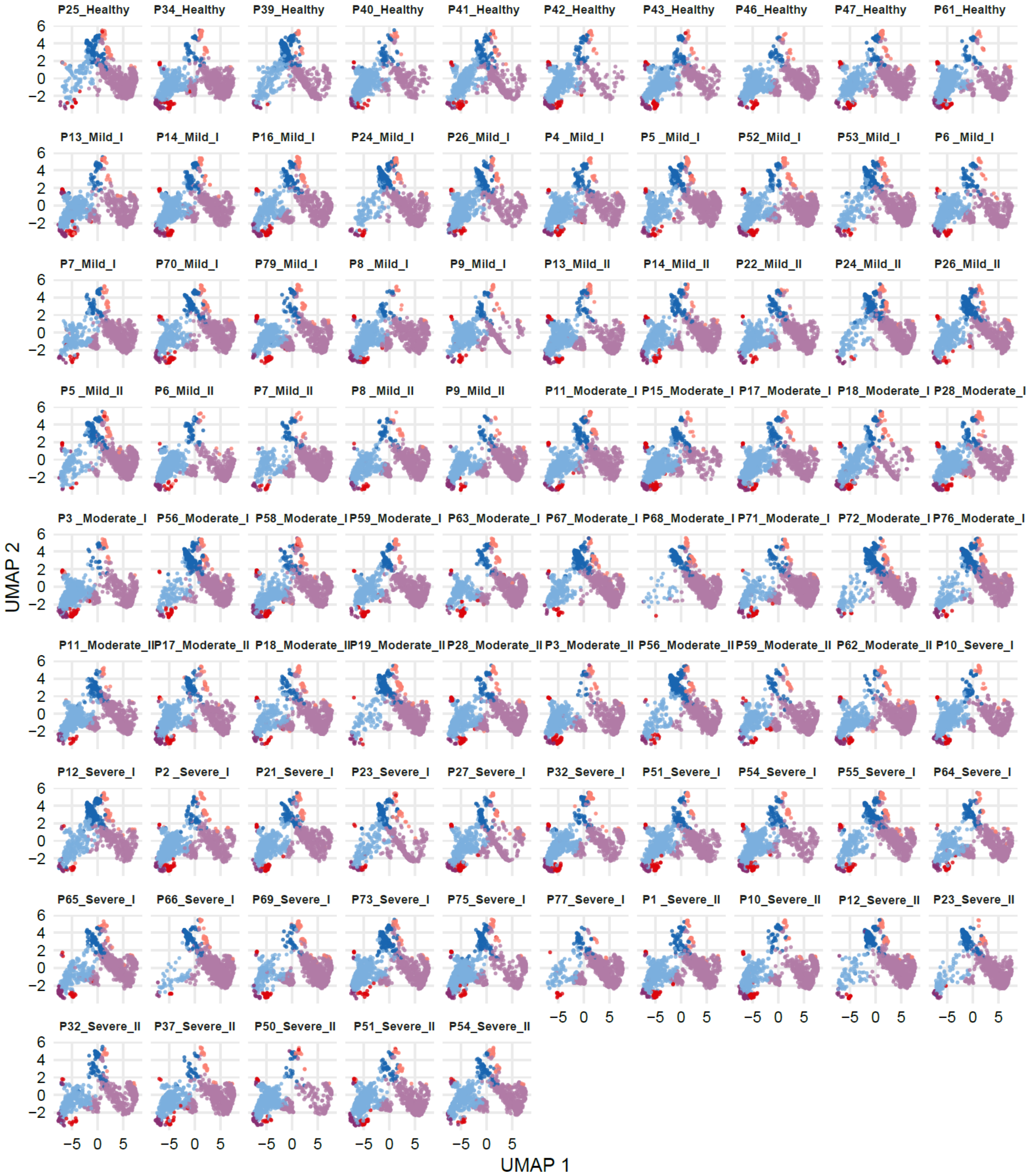

Supplementary Figure 6

Projection of CD4+ Treg UMAP graphs stratified by patient sample showing the FlowSOM clusters: : patient (P) number, Healthy control, COVID-19 severity (Mild, Moderate, Severe), and time-point I or II are given

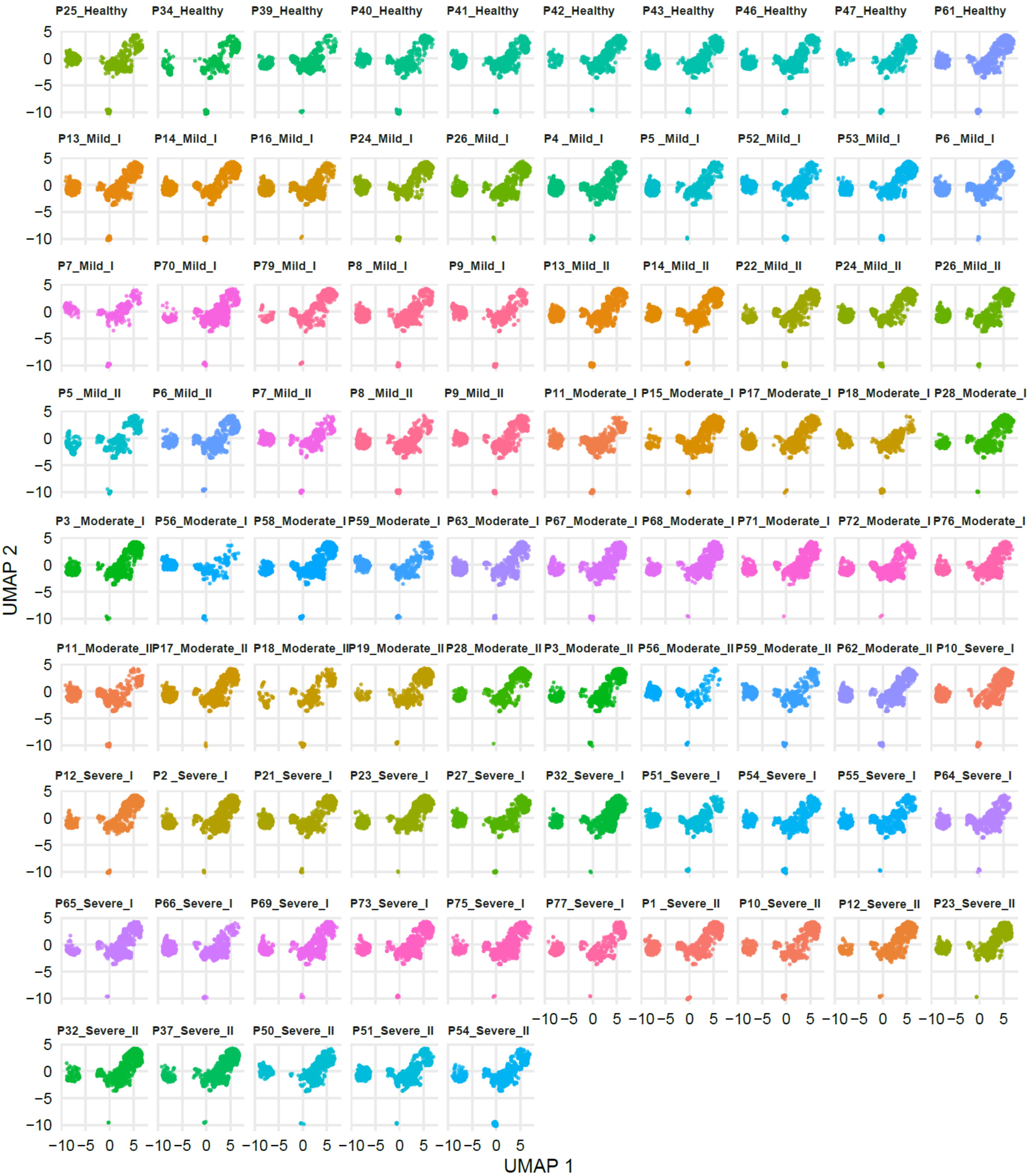

Supplementary Figure 7

CD8+ UMAP graphs stratified by patient sample: patient (P) number, Healthy control, COVID-19 severity (Mild, Moderate, Severe), and time-point I or II are given.

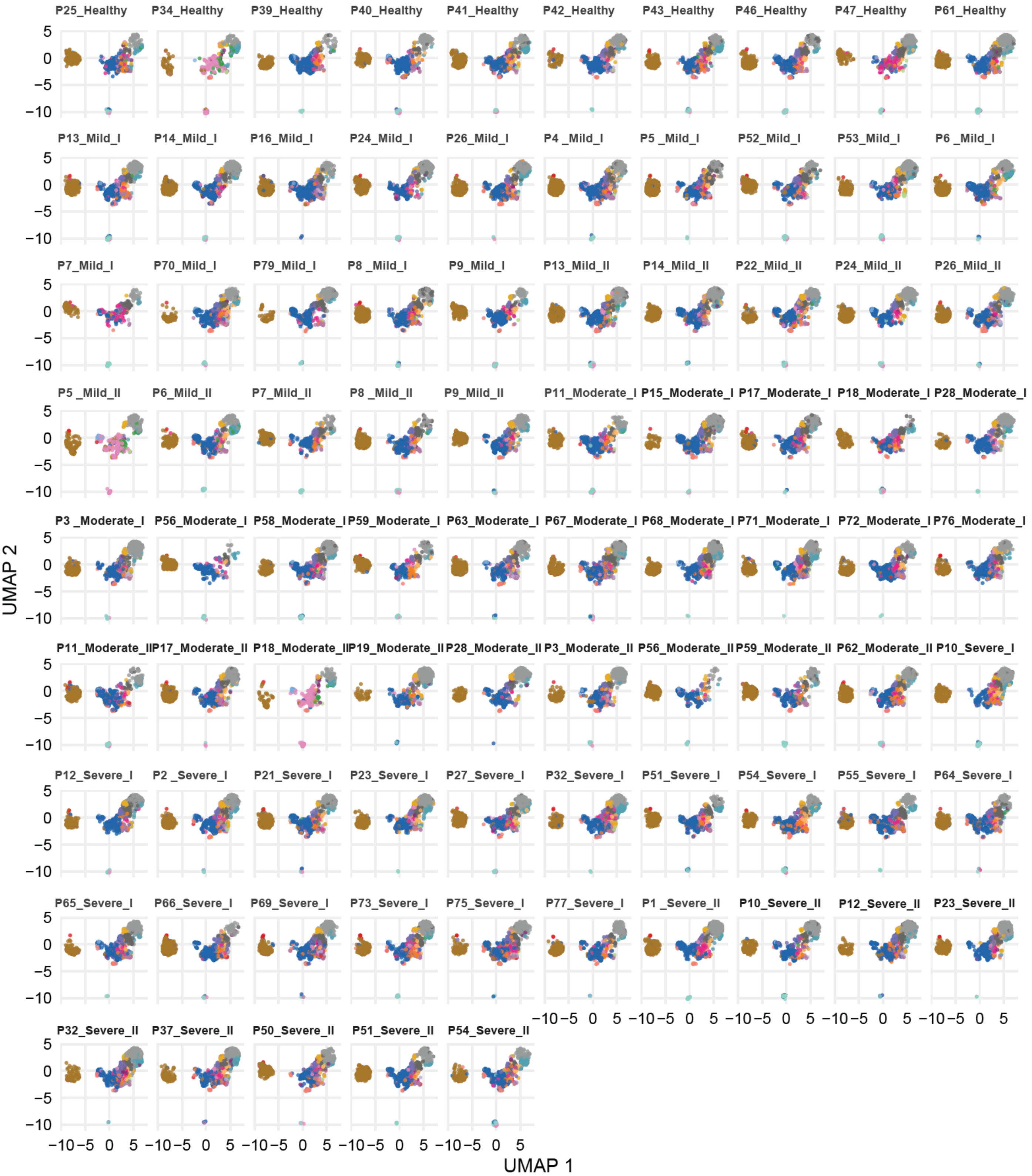

Supplementary Figure 8

Projection of CD8+ UMAP graphs stratified by patient sample showing the FlowSOM clusters: : patient (P) number, Healthy control, COVID-19 severity (Mild, Moderate, Severe), and time-point I or II are given

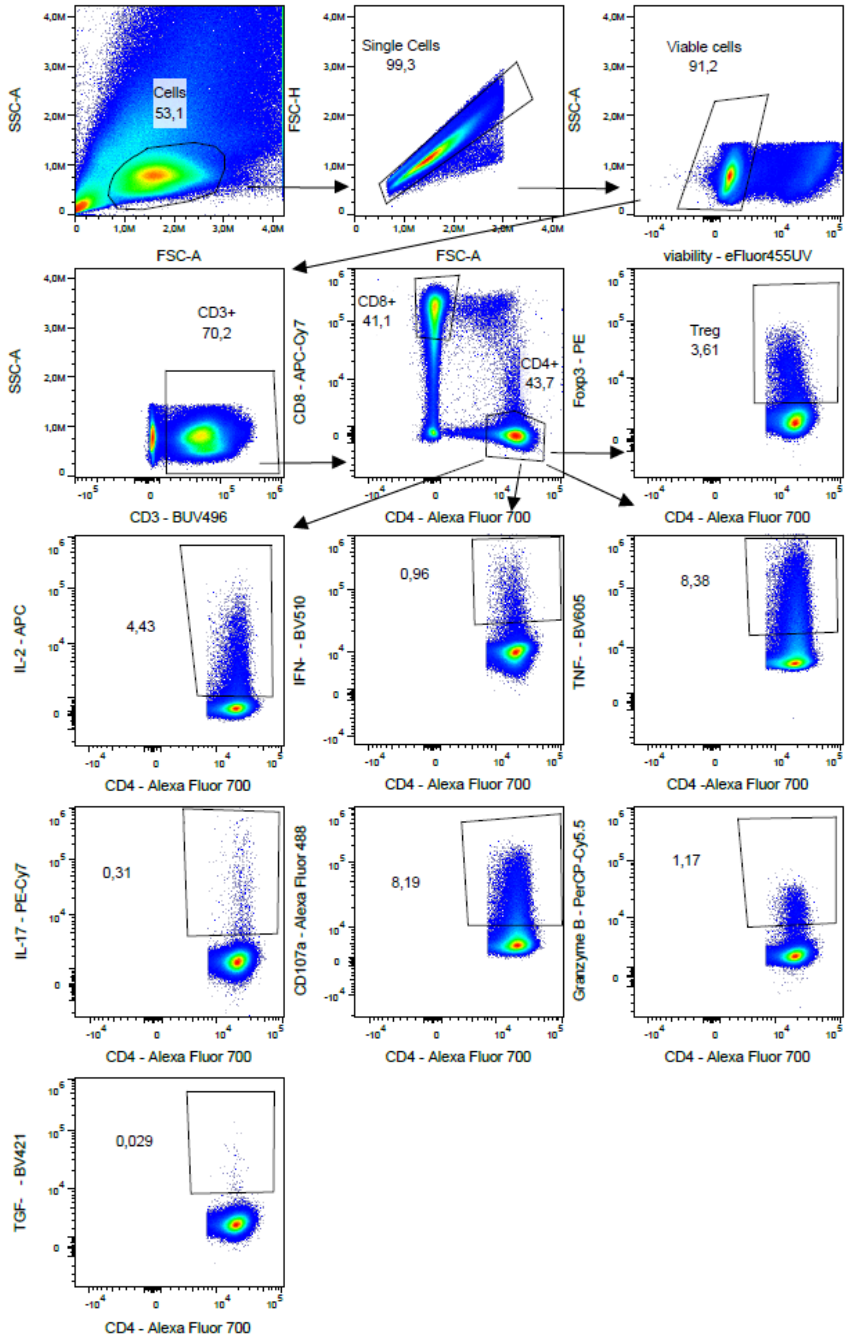

Supplementary Figure 9

The gating strategy that was used to identify cytokines production by CD4+ and CD8+ T as well as Treg cells after stimulation with anti-CD3/CD28. Gating of TGF-β BV421, IL-17 PE-Cy7, TNF-α BV605, IFN-ɣ BV510, IL-2 APC and granzyme-B PerCP-Cy5.5 for CD4+ T cells is shown. Gates for cytokines and other mediators present in CD8+ T cells and Tregs were set up in a similar way.

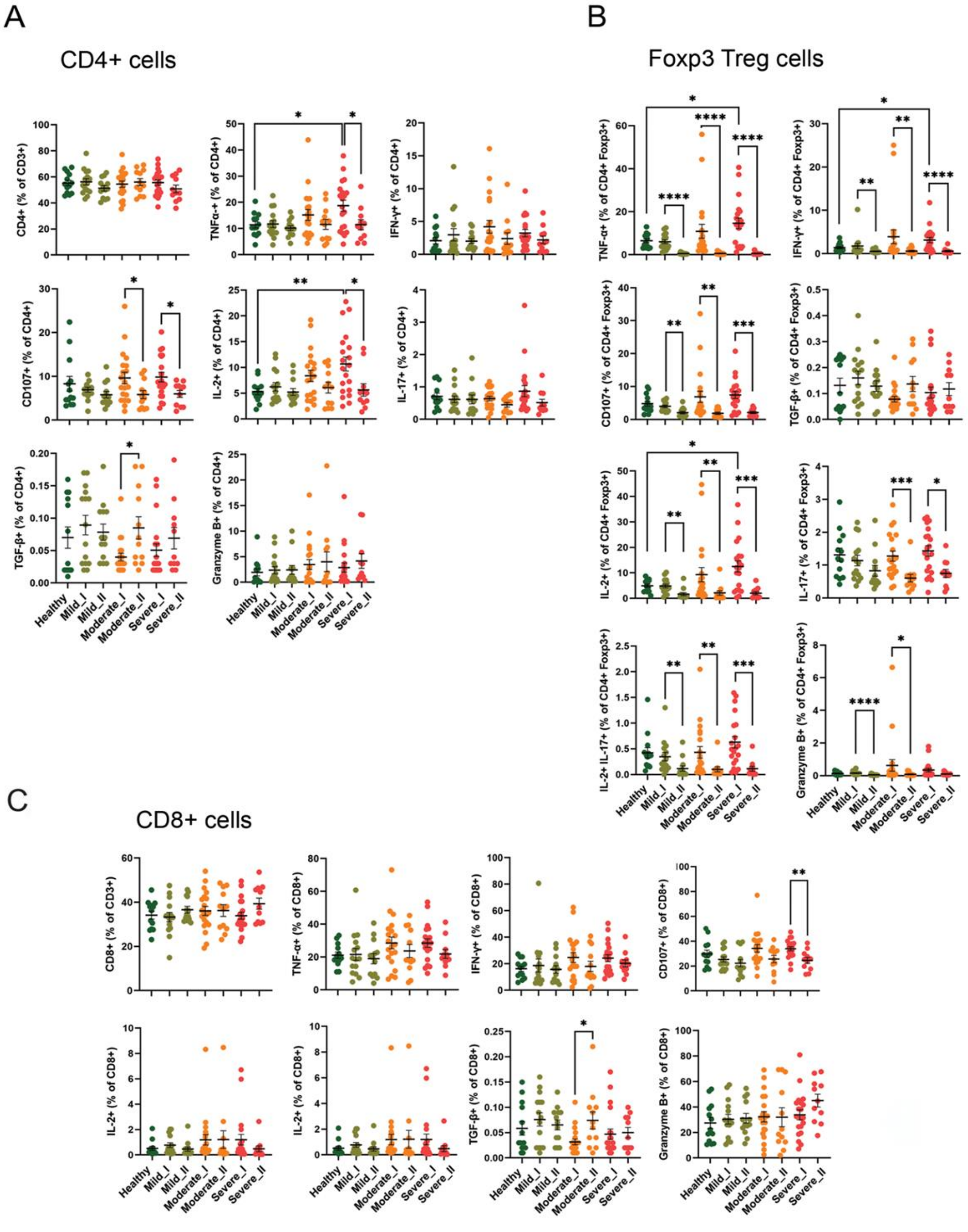

Supplementary Figure 10.

Cytokine production by CD4+, CD4+Treg and CD8+ cells after *in vitro* stimulation by anti-CD3/CD28. A. Percentage of CD4+ cells among CD3+ cells and comparison between the total production of TNF-α, IFN-ɣ, CD107a, IL-2, IL-17, TGF-β and granzyme-B by CD4+ T cells isolated from healthy donors and mild, moderate and severe COVID-19 convalescents analysed at time I and time II. **B.** Comparison between the total production of TNF-α, IFN-ɣ, CD107a, IL-2, IL-17 and granzyme-B by Treg cells isolated from healthy donors and mild, moderate and severe COVID-19 convalescents analysed at time I and time II. **C.** Percentage of CD8+ cells among CD3+ cells and comparison between the total production of TNF-α, IFN-ɣ, CD107a, IL-2, IL-17, TGF-β and granzyme-B by CD8+ T cells isolated from healthy donors and mild, moderate and severe COVID-19 convalescents analysed at time I and time II. Data represent individual values from controls and patients, mean (centre bar) ± SEM (upper and lower bars). Statistical analysis by two-sided Mann–Whitney nonparametric test; if not indicated, p value is not significant.
